# Nuclear genome sequence of the plastid-lacking cryptomonad *Goniomonas avonlea* provides insights into the evolution of secondary plastids

**DOI:** 10.1186/s12915-018-0593-5

**Published:** 2018-11-28

**Authors:** Ugo Cenci, Shannon J. Sibbald, Bruce A. Curtis, Ryoma Kamikawa, Laura Eme, Daniel Moog, Bernard Henrissat, Eric Maréchal, Malika Chabi, Christophe Djemiel, Andrew J. Roger, Eunsoo Kim, John M. Archibald

**Affiliations:** 1 Department of Biochemistry and Molecular Biology, Dalhousie University, Halifax, Nova Scotia B3H 4R2 Canada; 2 Centre for Comparative Genomics and Evolutionary Bioinformatics, Dalhousie University, Halifax, Nova Scotia Canada; 3 Graduate School of Human and Environmental Studies, Kyoto University, Kyoto, Kyoto 606-8501 Japan; 4 Architecture et Fonction des Macromolécules Biologiques (AFMB), CNRS, Université Aix-Marseille, 163 Avenue de Luminy, 13288 Marseille, France; 5 INRA, USC 1408 AFMB, 13288 Marseille, France; 6 Department of Biological Sciences, King Abdulaziz University, Jeddah, 21589 Saudi Arabia; 7 Laboratoire de Physiologie Cellulaire et Végétale, CNRS, CEA, INRA, Université Grenoble Alpes, Institut de Biosciences et Biotechnologies de Grenoble, CEA-Grenoble, 17 rue des Martyrs, 38000 Grenoble, France; 8 Present address: UMR 8576 – Unité de glycobiologie structurale et fonctionnelle, Université Lille 1, 59650 Villeneuve d’Ascq, France; 9 Canadian Institute for Advanced Research, Program in Integrated Microbial Biodiversity, Toronto, Ontario Canada; 10 Division of Invertebrate Zoology & Sackler Institute for Comparative Genomics, American Museum of Natural History, Central Park West at 79 Street, New York, NY 10024 USA; 11 Present address: Department of Cell and Molecular Biology, Science for Life Laboratory, Uppsala University, SE-75123 Uppsala, Sweden; 12 Present address: Laboratory for Cell Biology, Philipps University Marburg, Karl-von-Frisch-Str. 8, 35043 Marburg, Germany

**Keywords:** Cryptomonads, Cryptophytes, Secondary endosymbiosis, Phylogenomics, Genome evolution

## Abstract

**Background:**

The evolution of photosynthesis has been a major driver in eukaryotic diversification. Eukaryotes have acquired plastids (chloroplasts) either directly via the engulfment and integration of a photosynthetic cyanobacterium (primary endosymbiosis) or indirectly by engulfing a photosynthetic eukaryote (secondary or tertiary endosymbiosis). The timing and frequency of secondary endosymbiosis during eukaryotic evolution is currently unclear but may be resolved in part by studying cryptomonads, a group of single-celled eukaryotes comprised of both photosynthetic and non-photosynthetic species. While cryptomonads such as *Guillardia theta* harbor a red algal-derived plastid of secondary endosymbiotic origin, members of the sister group Goniomonadea lack plastids. Here, we present the genome of *Goniomonas avonlea*—the first for any goniomonad—to address whether Goniomonadea are ancestrally non-photosynthetic or whether they lost a plastid secondarily.

**Results:**

We sequenced the nuclear and mitochondrial genomes of *Goniomonas avonlea* and carried out a comparative analysis of *Go. avonlea*, *Gu. theta*, and other cryptomonads. The *Go. avonlea* genome assembly is ~ 92 Mbp in size, with 33,470 predicted protein-coding genes. Interestingly, some metabolic pathways (e.g., fatty acid biosynthesis) predicted to occur in the plastid and periplastidal compartment of *Gu. theta* appear to operate in the cytoplasm of *Go. avonlea*, suggesting that metabolic redundancies were generated during the course of secondary plastid integration. Other cytosolic pathways found in *Go. avonlea* are not found in *Gu. theta*, suggesting secondary loss in *Gu. theta* and other plastid-bearing cryptomonads. Phylogenetic analyses revealed no evidence for algal endosymbiont-derived genes in the *Go. avonlea* genome. Phylogenomic analyses point to a specific relationship between Cryptista (to which cryptomonads belong) and Archaeplastida.

**Conclusion:**

We found no convincing genomic or phylogenomic evidence that *Go. avonlea* evolved from a secondary red algal plastid-bearing ancestor, consistent with goniomonads being ancestrally non-photosynthetic eukaryotes. The *Go. avonlea* genome sheds light on the physiology of heterotrophic cryptomonads and serves as an important reference point for studying the metabolic “rewiring” that took place during secondary plastid integration in the ancestor of modern-day Cryptophyceae.

**Electronic supplementary material:**

The online version of this article (10.1186/s12915-018-0593-5) contains supplementary material, which is available to authorized users.

## Background

The acquisition of photosynthesis in eukaryotes can be traced back to a primary endosymbiosis in which a eukaryotic host engulfed and assimilated a photosynthetic cyanobacterium, which ultimately became the plastid (chloroplast) [1, 2]. Canonical “primary” plastids are surrounded by two membranes and are generally thought to have evolved on a single occasion in the common ancestor of Archaeplastida, a tripartite eukaryotic “supergroup” comprised of Viridiplantae (also known as Chloroplastida), Rhodophyta (Rhodophyceae), and Glaucophyta [3–5]. Eukaryotes have also acquired photosynthesis indirectly on multiple occasions via “secondary” (i.e., eukaryote-eukaryote) endosymbiosis. Indeed, secondary (and in some cases tertiary) endosymbiosis is thought to have given rise to plastids scattered amongst the stramenopiles, alveolates, rhizarians, euglenozoans, haptophytes, and cryptomonads [6]. The latter lineage is divided into two clades, the plastid-bearing, mostly photosynthetic Cryptophyceae and the heterotrophic Goniomonadea. The evolutionary distinctness of these two clades makes for an interesting case study with which to understand the transition from a plastid-lacking eukaryote to a photosynthetic, secondary plastid-bearing organism.

*Guillardia theta* and the recently described *Goniomonas avonlea* [7] are representatives of plastid-bearing and plastid-lacking cryptomonads [5, 8], respectively. Together with several paraphyletic plastid-lacking lineages, including katablepharids and *Palpitomonas*, cryptomonads constitute a clade known as Cryptista [9, 10]. The position of Cryptista on the eukaryotic tree of life is a point of contention. Some phylogenomic studies have placed it sister to Haptophyta (e.g., [11]), with the Cryptista-Haptophyta clade itself branching either next to the SAR supergroup (Stramenopiles, Alveolata, Rhizaria; e.g., [12]) or the Archaeplastida (e.g., [13]). Other studies have suggested that Cryptista and Haptophyta are not specifically related, with the former branching within the Archaeplastida [14]. Our knowledge of Cryptista and their evolutionary history has suffered from a paucity of genomic data [15]. Only one species, *Gu. theta*, which has been studied mainly for its plastid and nucleomorph (the vestigial nucleus of the red alga acquired by secondary endosymbiosis) [16, 17] has had its nuclear genome sequenced [18]. The diversity of plastid-lacking species within cryptomonads and, more broadly, Cryptista, has received relatively little attention.

The transition from a heterotrophic, aplastidic cell to a plastid-bearing one is associated with the acquisition of a wide range of metabolic capabilities, such as photosynthesis and novel amino acid biosynthetic capacities [19]. The acquisition of photosynthesis and carbon fixation by a heterotrophic protist impacts the regulation of many of its metabolic pathways [20]. In addition, pathways operating in different subcellular compartments can become partially or completely redundant. This allows the organism to tinker with the regulation of pathways that may be adapted to a particular cellular compartment and/or set of metabolites. Endosymbiosis can also give rise to mosaic metabolic pathways comprised of enzymes with different evolutionary origins [18, 21]. Proteins may be derived from the host, from the endosymbiont (both primary and secondary), or as a result of lateral gene transfer (LGT) from different prokaryotic and eukaryotic organisms. Understanding how cells adapt from living in a solitary state to having another organism within it is fundamental to understanding the evolution of plastid-bearing organisms.

We have sequenced the nuclear genome and transcriptome of the plastid-lacking goniomonad *Go. avonlea* CCMP3327 [7] with the goal of shedding light on its physiology and, more generally, the metabolic transformation that accompanied the transition from heterotrophy to phototrophy in its plastid-bearing sister taxa. Using comparative genomics and phylogenomics, we found little evidence for a photosynthetic ancestry in *Go. avonlea* and show that the acquisition of a plastid in an ancestor of present-day Cryptophyceae resulted in extensive reshuffling of metabolic pathways. Annotation of carbohydrate-active enzymes (CAZymes) [22] including glycosyltransferases (GTs), glycoside hydrolases (GHs), polysaccharide lyases (PLs), and carbohydrate esterases (CEs) allows us to make several predictions about the lifestyle of *Go. avonlea* and other goniomonads, including the possibility that they feed on multiple organisms, including eukaryotic algae.

## Methods

### Cell culture, nucleic acid preparation, and genome sequencing

*Goniomonas avonlea* CCMP3327 was grown in ESM medium [23] supplemented with ATCC’s 1525 Seawater 802 medium. One day prior to harvesting, a dose of Penicillin-Streptomycin-Neomycin antibiotic mixture (Thermo Fisher cat #15640055) was administered in order to reduce the number of co-cultured bacteria. Cells were harvested in two steps. First, liquid culture was filtered through a 2-μm pore-sized polycarbonate membrane disc in order to deplete bacterial cells; the remaining protist cells were re-suspended in artificial seawater and transferred to a falcon tube. Cells were pelleted by centrifugation at 3000 RCF for 8 min. DNA was extracted using a PureLink® Genomic DNA Kit (Thermo Fisher Scientific, cat# K182001). For RNA preparation, cells were lysed and phase-separated using TRIzol™ reagent (Thermo Fisher Scientific, cat #15596018), followed by the use of the RNeasy Mini Kit (QIAGEN, cat #74104) for precipitation, washes, and elution.

DNA and RNA samples were sent to Génome Québec and the Beijing Genomics Institute (BGI) for library preparation and sequencing on the Illumina HiSeq2000 platform. At Génome Québec, genomic data were generated from a short-insert library, while 2 kb and 6 kb mate pair libraries were sequenced at the BGI. A total of 209,988,904, 12,244,898, and 35,906,071 forward and reverse reads, up to 100 bp in length, were generated for the short-insert, 2 kb, and 6 kb mate pair libraries, respectively. For the transcriptome, 62,428,409 forward and reverse reads were sequenced, up to 100 bp in length, from a library prepared with the TruSeq protocol at Génome Québec.

### Genome assembly, gene prediction, and quality control

Transcriptome reads were quality trimmed using Trimmomatic [24] and assembled de novo with Trinity [25]. The genome was assembled with ALLPATH-LG [26], Abyss [27], Minimus2 [28] and Ray [29]. We considered the N50 values of the two “best” assemblies (Abyss and ALLPATHS-LG) and used BOWTIE2 [30] coupled with ALE [31] and CGAL [32] to evaluate which assembly was optimal for our purposes. The ALLPATHS-LG assembly was selected and subjected to a blastn analysis [33]; contigs with bacterial hits with E-values lower than 1e^− 50^ were considered bacterial and removed. We then predicted protein-coding genes using both Augustus [34, 35] and PASA [36], which allowed correction of gene models using transcriptome data. To further reduce the chance of bacterial contamination, we carried out blastp searches of our predicted proteins against NCBI nr (ftp://ftp.ncbi.nlm.nih.gov/blast/db/) and the Marine Microbial Eukaryote Transcriptome Sequencing Project database (ftp://ftp.imicrobe.us/camera/) [37]. For each protein sequence, we took the first 10 hits and considered the protein to be from Eukaryota if > 60% of the hits were eukaryotic, or bacterial or archaeal if > 60% of the hits were to Bacteria or Archaea. Sequences that did not pass either threshold were assessed manually. In such cases, genes were mapped to their contigs and if > 60% of the gene models on the contig were eukaryotic, we considered the contig to be derived from the *Goniomonas avonlea* nuclear genome.

To identify as many protein-coding genes as possible and to ensure their full length, we predicted all ORFs from the *Go. avonlea* transcriptome, generating six frame translations for each transcript. From the pool of possible ORFs, we took the four longest translations and blasted them against the nr and MMETSP databases. All translated transcripts with a hit below 1e^− 05^ were kept. To these transcriptome-derived sequences, we added protein sequences predicted from the genomic data using Augustus and PASA. The added sequences were those that had a hit < 1e^− 05^ against the nr or MMETSP databases and that did not already match proteins from the transcriptome dataset with sequence identity > 90%. The resulting set of 18,429 protein coding sequences, used for all subsequent analyses, represents the union of predicted gene models and predicted ORFs and represents a refined set of protein sequences demonstrably from *Goniomonas* and likely to have homologs in other organisms.

We assessed genome “completeness” using BUSCO (v1; [38]), which is based on a set of 429 protein-coding genes purported to be universally present in eukaryotes as single copies [37, 38]. The 18,429 *Go. avonlea* proteins were analyzed; the BUSCO results were compared to those obtained for *Gu. theta* and the amoebozoan *Dictyostelium discoideum*.

### Orthologous protein annotation and KOG classification

For *Go. avonlea*, *Gu. theta*, *Bigelowiella natans*, *Emiliania huxleyi*, *Adineta vaga*, and *Arabidopsis thaliana*, we clustered orthologous sequences using OrthoVenn (http://www.bioinfogenome.net/OrthoVenn/) [39], with E-value and inflation value settings at 1e^− 5^ and 1.5, respectively. In addition, we compared the size and diversity of KOG functional categories (EuKaryotic Orthologous Groups) inferred from both the *Go. avonlea* and *Gu. theta* genomes using the WebMGA server (http://weizhong-lab.ucsd.edu/webMGA/server/kog/) with an E-value cut-off of 1e^− 03^ [40].

### Protein annotation and sub-cellular localization prediction

Protein annotation was performed using KOBAS [41]; proteins that were not annotated using this approach were analyzed using GhostKOALA [42]. Annotations were then used to generate KEGG metabolic pathway maps (http://www.genome.jp/kegg/tool/map_pathway.html) [43].

In order to predict the sub-cellular locations of *Go. avonlea* proteins, we first selected 13,508 sequences inferred from our genome assembly that (i) start with a methionine and (ii) match the amino (N)-termini of proteins in our final set of 18,429 proteins, reasoning that proteins derived from our genomic (and not transcriptomic) data were more likely to possess full length N termini. Given the uncertainty of whether or not *Go. avonlea* and other goniomonads evolved from a plastid-bearing ancestor, we carried out different predictions using a combination of PredSL [44], TargetP [45], and Predotar [46] under the “plant” and “non-plant” modes (Additional file 1). Considering the formal possibility that *Go. avonlea* could, like *Gu. theta*, have a plastid acquired by secondary endosymbiosis, we used SignalP 4.1 [47, 48] coupled with ASAFind [49] to predict periplastidial compartment (PPC) and plastid proteins. For *Gu. theta*, the predicted protein coding gene set from Curtis et al. [18] was used, as were the signal peptide predictions for the purposes of comparison with *Go. avonlea*.

### Annotation of carbohydrate-active enzymes (CAZymes)

We performed a manual annotation of CAZymes [22] using a mix of BLAST [33] and HMM searches [50], similar to that done previously for *Gu. theta* [18]. To assess the similarity between the two species across CAZyme families, we generated heat maps derived from an average linkage hierarchical clustering based on Bray-Curtis dissimilarity matrix distances and Ward’s method [51–54]. The phylogenetic heat maps were generated with Rstudio software (https://www.rstudio.com/) using vegan in the R package (http://cc.oulu.fi/~jarioksa/softhelp/vegan/html/vegdist.html) [55] with *vegdist* and *hclust* commands.

### Phylogenetic analysis of “algal” genes

We sought to identify putative algal-derived homologs in the *Go. avonlea* genome by comparing its gene/protein set to that of *Gu. theta* and other algae. More specifically, we used blastp to search a custom database of 508 proteins predicted to be the product of endosymbiotic gene transfer (EGT) in *Gu. theta* [18]. For each *Go. avonlea* protein with a significant hit to this database (E-value < 1e^− 10^), we used DIAMOND with the “more sensitive” option [56] to retrieve the top 2000 homologs above an E-value cut-off of 1e^− 10^ from the nr and MMETSP [37] databases. Paralogs in the *Go. avonlea* candidate-EGT set were then identified by pairwise comparison of DIAMOND outputs; if two queries had > 50% overlap in hits they were considered paralogous and merged. Candidate *Go. avonlea* EGTs were annotated using InterPro [57] and their subcellular localizations were predicted as above.

Single-gene/protein trees were generated from alignments initially produced using MAFFT (version 7.205 [58]). Ambiguously aligned regions were removed using BMGE (version 1.1 [59]) with the BLOSUM30 scoring matrix and a block size of 4; trimmed alignments shorter than 50 amino acids were discarded. For the remaining candidates, an approximately maximum likelihood phylogeny was generated using FastTree [60] and used in an in-house tree-trimming script to reduce taxonomic redundancy in each dataset. Reduced datasets were then re-aligned using MAFFT-linsi (version 7.205 [58]), trimmed as above, and filtered to discard alignments shorter than 70 amino acids. Maximum-likelihood (ML) phylogenies were inferred for each remaining candidate in IQ-TREE (Version 1.5.5 [61]) under the LG4X substitution model [62] with 1000 ultra-fast bootstrap approximations (UFboot) [63]. The resulting trees were manually evaluated and sorted based on the topology of the *Go. avonlea* and *Gu. theta* proteins in relation to each other and to sequences from various combinations of primary and secondary plastid-bearing photosynthetic lineages.

Additional genes/proteins of particular interest (for example, the CAZymes glycosyltransferase 28 and glucan water dikinase) were investigated on a case-by-case basis using a similar approach as above and as described in several other studies [64, 65]. In these cases, homologs to predicted *Go. avonlea* genes/proteins were identified in various additional genomic/transcriptomic datasets, sequence redundancy was reduced using a combination of manual inspection and an automated treetrimmer analysis [66] and “final” alignments were produced with MUSCLE [67].

### Phylogenomics

To investigate the phylogenetic position of Cryptista on the eukaryotic tree of life, a 250-marker gene, un-aligned dataset consisting of 150 operational taxonomic units (OTUs) corresponding to Burki et al. [14] was obtained from the Dryad Digital Repository [68]. The number of OTUs was systematically reduced to decrease the complexity of phylogenetic analyses while maintaining taxonomic diversity and minimizing missing data. Proteins predicted from the *Go. avonlea* transcriptome data were added to the dataset to increase marker gene coverage for the Goniomonadea. *Go. avonlea* homologs were identified using blastp with any Cryptista sequence (if available) or the first sequence in the marker gene set as the query; the best hit (E-value < 1e^− 10^) in *Go. avonlea* was added to the dataset. Each marker gene/protein was aligned using MAFFT-linsi (version 7.205; [58]), and ambiguously aligned sites were removed using BMGE (version 1.1 [59]). Single gene trees were inferred using ML methods in IQTREE (Version 1.4.3 [61]) under the substitution model LG4X [62] with 1000 UFboot [63] and manually inspected to identify any obvious potential artifacts (e.g., long branch attractions). Marker genes/proteins were then realigned and subject to block removal prior to concatenation. The resulting supermatrix was used to infer a ML phylogeny in IQTREE (Version 1.4.3 [61]) using the model LG + C60 + F + PMSF [69] (selected according to the Bayesian Information Criterion (BIC) based on the outcome of a model test implemented in IQTREE [70]) with 100 standard bootstrap iterations.

In order to further investigate the phylogenetic position of Cryptista, phylogenetic trees were inferred (as above) based on modified versions of the supermatrix in which (i) sequences from plastid-bearing cryptistan taxa (i.e., Cryptophyceae) were removed from the dataset and (ii) PhyloMCOA [71] was used to identify and remove discordant genes in each OTU based on phylogenetic positioning across single-gene trees (using nodal distances) and multiple co-inertia analysis (MCOA). To evaluate the significance of differences in branch support, the standard error of bootstrap values was used with a 95% confidence interval. To explore alternative signals emerging from Cryptista in the marker gene dataset, 183 of the 250 genes (those that contained a homolog in *Go. avonlea* and at least one other cryptomonad) were randomly partitioned into four equally sized bins. Each bin of marker genes was concatenated and used to infer a phylogeny based on ML methods in IQTREE (Version 1.4.3; [61]) under the model LG + C20 + F. This process was repeated 25 times, resulting in 100 randomly generated marker gene subset trees; for each tree, the phylogenetic position of Cryptista was manually evaluated.

## Results and discussion

### The *Goniomonas avonlea* nuclear and mitochondrial genomes

We sequenced the *Go. avonlea* nuclear genome to a depth of ~ 24× coverage. In part due to the presence of repetitive sequences, the assembly is highly fragmented. For the final assembly, we retained contigs at least 500 bps in length resulting in 31,852 contigs (N50 = 3831) totalling 91.5 Mb and a GC content of 55.2% (Table 1). From our initial assembly, 33,470 genes were predicted; further investigation revealed that this number was artificially inflated due to assembly fragmentation. We thus merged protein-coding genes predicted from the genome with those inferred from transcriptome data (see “Methods”). This resulted in a set of 18,429 non-redundant protein-coding genes. When analyzed with BUSCO [38], our protein sequence data set was predicted to be 69% “complete,” 20% “fragmented,” and 9.7% “missing”; this is comparable to the previously sequenced genome of *Gu. theta*, which was inferred to be 78% “complete,” 12% “fragmented,” and 8.8% “missing” (Additional file 2: Table S1). This suggests that despite the level of assembly fragmentation, the *Go. avonlea* protein coding gene set is similar in terms of completeness to that of *Gu. theta*; conclusions about the presence/absence of metabolic pathways in the two genomes (see below) are probably not adversely affected by missing data. It should also be noted that in general such analyses are limited by a distinct lack of knowledge of cryptomonads and their large phylogenetic distance from the organisms used to create the BUSCO reference dataset. For reference, the well-annotated genome of the amoebozoan protist *D. discoideum* is inferred to be 5.1% “missing” using BUSCO (Additional file 2: Table S1).

**Table 1:** General genome features for *Guillardia theta* and *Goniomonas avonlea*

	*Guillardia theta*	*Goniomonas avonlea*
Assembly size	87.1 Mb	91.5 Mb
# scaffolds	669	31,852
# contigs	5126	31,852
N50 scaffolds	40,445 bp	3831 bp
GC%	52.9	55.2
# of protein coding genes	24,822	33,470
# of introns	132,885	112,740
Percentage of genes with introns	79%	84%
Number of forward genes	12,482	16,836
Number of reverse genes	12,441	16,638
Average size of gene (nt)	1863	1626
Intron size average (nt)	106	171
Intron size mode (percent of total)	47 (5.1%)	46 (4.7%)
Average # of introns per gene	5.3	3.5

**Fig. 1:**
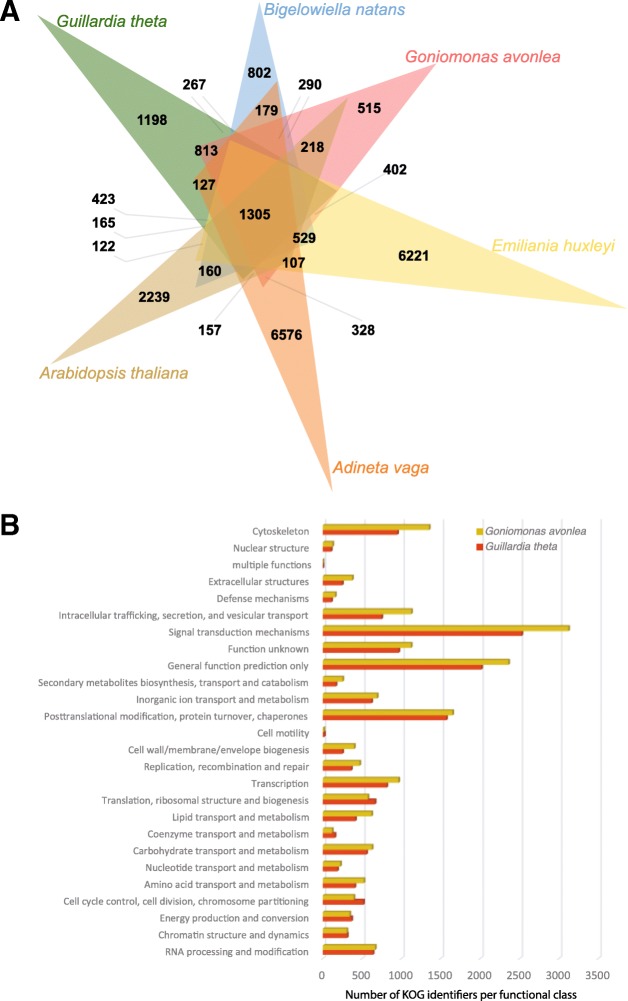
Comparative genomics of Goniomonadea, Cryptophyceae, and other eukaryotes. **a** Venn diagram showing orthologous clusters shared between the goniomonad *Goniomonas avonlea* (red), the cryptophyte *Guillardia theta* (green), the rhizarian *Bigelowiella natans* (blue), the haptophyte *Emiliania huxleyi* (yellow), the opisthokont *Adineta vaga* (orange), and the land plant *Arabidopsis thaliana* (brown). *Go. avonlea* shares 4321 families with *Gu*. *theta*, higher than is shared with other eukaryotes (*B. natans* (3647), *E. huxleyi* (3441), *A. vaga* (3173), *A. thaliana* (2955)). **b** KOG classification of proteins in *Go*. *avonlea* (brown) and *Gu. theta* (red). Within most functional categories, the number of proteins in the two organisms is similar. However, *Go. avonlea* possesses more proteins in some categories, in particular the cytoskeleton and the intracellular trafficking, secretion, and vesicular transport families

Analysis of orthologous groups of proteins shared between the cryptomonads *Go. avonlea* and *Gu. theta*, as well as the rhizarian *Bigelowiella natans*, the haptophyte *Emiliania huxleyi*, the rotifer *Adineta vaga*, and the model land plant *Arabidopsis thaliana*, shows that, as expected, *Go*. *avonlea* and *Gu*. *theta* share more orthologous protein families with each other (4321 in total) than they do with other organisms (*Go. avonlea* shares 3647, 3441, 3173, and 2955 protein families with *B. natans*, *E. huxleyi*, *A. vaga*, and *A. thaliana*, respectively; Fig. 1a). Nevertheless, comparison of KOGs present in both *Go*. *avonlea* and *Gu*. *theta* reveals differences in the size and complexity of certain KOG functional categories (Fig. 1b). For example, KOG categories corresponding to cytoskeleton and intracellular trafficking, secretion, and vesicular transport are more abundant in *Go*. *avonlea*. Such differences may in part be due to the obligate phagotrophic lifestyle of *Go*. *avonlea* (see below). In contrast, *Gu. theta* appears somewhat enriched (relative to *Go. avonlea*) in functions associated with translation, ribosomal structure and biogenesis, as well as cell cycle control/division and chromosome partitioning (Fig. 1b).

We also sequenced and assembled the mitochondrial genome of *Go. avonlea*, which at 41.2 kb in size is similar to the circular mapping genomes of cryptophytes (when repeated sequences are excluded; e.g., [72, 73]) and the linear mtDNA of *Palpitomonas bilix* [74]. There are also interesting similarities amongst these mtDNAs in terms of gene content (Additional file 2: Figure S1). When compared to the mitochondrial genomes of other eukaryotic lineages, all members of Cryptista considered here share very similar gene repertoires, especially for complexes I-V of the electron transport chain (Additional file 2: Figure S1): they all have *nad1–4*, *4L*, *5–11*, *sdh3*, *4*, *cob*, *cox1–3*, *atp1*, *3*, *4*, *6*, *8*, and *9*. That said, *Go. avonlea* and *P. bilix* share an *rpl2* gene that is not present in cryptophyte mtDNAs, and all sequenced cryptophyte mtDNAs possess an *rps2* gene [73] that is not present in *Go. avonlea* or *P. bilix*. *Go. avonlea* mtDNA also lacks the *tatA* and *tatC* genes found in other Cryptista mtDNAs, which encode components of the twin arginine translocator. Of particular note, like cryptophytes, *Go. avonlea* lacks the mitochondrial *ccmA*, *B*, *C*, and *F* genes recently found in the mtDNA of *P. bilix* [74]. These genes encode a bacterial-type cytochrome *c* maturation system (“system I”); our data support the hypothesis that goniomonads use a nucleus-encoded holocytochrome c synthase (HCCS) system (i.e., “system III”). We searched for, and found, the HCCS gene in the nuclear genome of *Go. avonlea* (comp53045_c0_seq2_6_ORF10_179 and comp39203_c0_seq2_6_ORF3_158). This confirms the authenticity of such genes found in transcriptome data from *Go. pacifica* and the katablepharid *Roombia* sp. NY0200 [74]. With its mitochondrial-encoded “system I” cytochrome *c* maturation system, *P. bilix* is thus an outlier amongst cryptistan protists, which raises interesting questions about how the type I and III systems evolved in these and other organisms (see Nishimura et al. [74] for discussion). All things considered, our mitochondrial genome analyses are consistent with phylogenomic data suggesting that although the organisms that comprise Cryptista are not closely related, they represent a monophyletic assemblage on the eukaryotic tree of life [9, 10, 14].

### *Goniomonas avonlea* does not have a plastid

On the basis of electron microscopy, *Go. avonlea* cells do not have any obvious plastid-like internal structures [7]. Nevertheless, with complete genome and transcriptome sequences in hand, we explored its predicted metabolic pathways as well as putative TOC-TIC proteins in an effort to detect any hint of evidence for a cryptic plastid—none was found (Additional file 2: Figure S2). Moreover, we predicted the sub-cellular locations of all of the *Go. avonlea* proteins under the following hypothetical scenarios: (i) the organism does not have a plastid, (ii) it has a cryptic plastid derived from primary endosymbiosis, or (iii) it has a cryptic plastid of secondary endosymbiotic origin. In short, we found no evidence supporting the presence of a plastid of primary or secondary endosymbiotic ancestry; hundreds of candidate proteins were identified using various search procedures (e.g., presence of bipartite *N-*terminal targeting sequences) but closer investigation revealed these to be false positives (Additional file 1 and Additional file 2: Figure S3). This is consistent with previous analyses performed on transcriptome data from *Goniomonas pacifica* [10] as well as microscopic observations of several *Goniomonas* strains, including *Go. avonlea* [7, 75, 76].

### Absence of endosymbiotically derived algal genes in *Goniomonas avonlea*

**Fig. 2:**
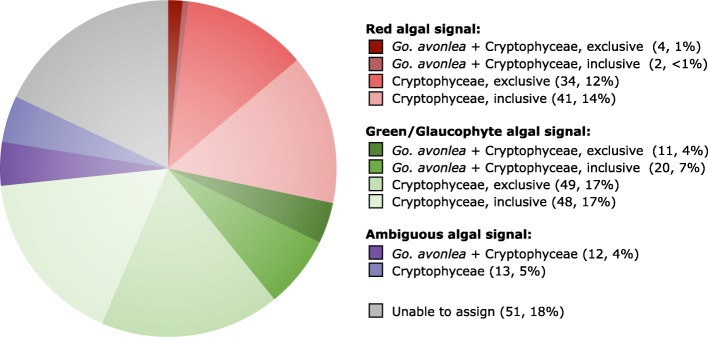
Algal genes in *Guillardia theta* and *Goniomonas avonlea*. The diagram shows the distribution of topologies observed in *Go. avonlea* homologs to 508 “algal” endosymbiotic gene transfers predicted by Curtis et al. [18] in *Gu. theta*. Phylogenies were evaluated and sorted based on the relative positioning of *Go. avonlea* and *Gu. theta* (and other Cryptophyceae) and their relationship to Archaeplastida lineages and secondarily photosynthetic taxa. An exclusive relationship indicates a direct relationship with an Archaeplastida lineage while an inclusive one indicates there are intervening secondarily photosynthetic taxa. A given topological pattern was only assigned if the corresponding UFboot support was greater than 80%. Of the 285 homologs identified in *Go. avonlea*, only six show an affinity to Cryptophyceae and red algae

**Fig. 3:**
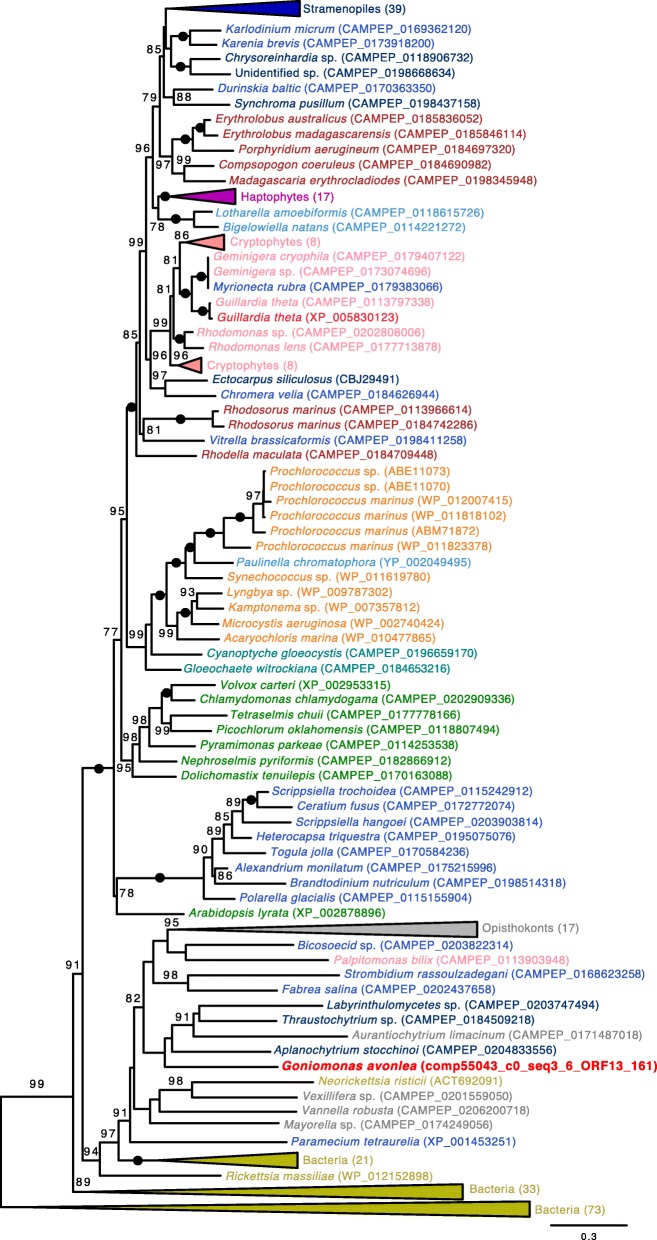
Maximum likelihood (ML) phylogeny of tryptophanyl-tRNA synthetase in diverse eukaryotes and prokaryotes. The tree was inferred under the model LG4X (with 100 standard bootstrap replicates) and shows an apparent red algal ancestry for homologs in Cryptophyceae but not in *Go. avonlea*. Eukaryotic OTUs are colored according to their known or predicted “supergroup” affinities with sequences from *Go. avonlea* and predicted *Gu. theta* EGTs [17] highlighted in bright red (Viridiplantae are in green, Glaucophyta are in turquoise, Rhodophyta are in dark red, Cyanobacteria are orange and other Bacteria are in gold, Cryptophyta are in pink, Haptophyta are in purple, Stramenopiles are in dark blue, Alveolata are in blue, Rhizaria are in light blue). The tree shown is midpoint rooted. Black dots indicate maximal support for particular nodes. When not maximal, only bootstrap support values > 70% are shown. The scale bar shows the inferred number of amino acid substitutions per site

Curtis et al. [18] identified 508 genes of probable endosymbiont (i.e., algal) ancestry in the *Gu. theta* nuclear genome. Many of these endosymbiotic gene transfers (EGTs) encode proteins that are predicted to have been repurposed and to function in the host cytosol of *Gu. theta* or other host-associated compartments; if *Go. avonlea* lost a red-algal-derived plastid secondarily, one might thus predict that at least some of these algal genes would still be present in its genome [18, 77]. Using sequence homology searches, we found that *Go. avonlea* has one or more homologs to 212 of the 508 *Gu. theta* EGT genes (285 *Go. avonlea* proteins in total). Manual investigation of the phylogenies of each of these 285 proteins (Fig. 2) revealed that only six show any obvious red algal signal in both Cryptophyceae (including *Gu. theta*) and *Go. avonlea*, none of which were predicted to be targeted to a plastid or function in plastid metabolism. In contrast, the Cryptophyceae showed a significant red-algal signal to the exclusion of *Go. avonlea* in 75 of these 285 phylogenies (e.g., tryptophanyl-tRNA synthetase; Fig. 3). Similar to the results of Curtis et al. [18], a large proportion of these trees were found to be ambiguous with respect to the nature of their algal signal. In some cases, the cryptophyte homologs branch closest to green or glaucophyte algae (31/285 and 97/285 trees where a *Go. avonlea* homolog branches with or without the predicted *Gu. theta* EGT in the phylogeny, respectively), while in others the primary algal lineage is entirely unclear (12/285 trees without a *Go. avonlea* homolog branching with the predicted cryptophyte EGT, 13/285 where a *Go. avonlea* homolog branches with the predicted cryptophyte EGT). However, given that the phylogenetic position of Cryptista relative to Archaeplastida and other eukaryotic supergroups is unclear [14], extreme caution is needed when considering these “green,” “glaucophyte” or ambiguous algal genes as bona fide EGTs, particularly in cases where obvious plastid targeting signals and/or plastid-associated functions are not observed [18]. Here, plastid-targeting signals and/or plastid-associated functions were not observed for any *Go. avonlea* homolog that branched with a *Gu. theta* predicted EGT showing a common “green,” “glaucophyte,” or ambiguous algal phylogenetic signal.

Is this small “red algal” footprint in the *Go. avonlea* genome (6/508 predicted algal EGTs in *Gu. theta*) meaningful? Comparing this signal to that observed against a control taxon (i.e., an unrelated amoebozoan with an unambiguous non-photosynthetic ancestry) allowed us to assess the expected signal due to background phylogenetic noise [77]. We found that *Go. avonlea* appeared sister to Amoebozoa in 24/285 single-gene trees, substantially higher than the red algal fraction. These analyses strongly suggest that the common red-algal footprint in *Go. avonlea* and *Gu. theta* is not significant, consistent with the lack of evidence for the existence of a cryptic plastid from microscopy and protein subcellular localization predictions. It is unclear to what extent our phylogenomic results are biased by taxonomic sampling; genome sequence data are presently stacked in favor of green algae and land plants over red algae [15]. It will thus be interesting to see whether the number of “red-algal” genes in Cryptophyceae (as well as other complex red algae-derived plastid bearing taxa) and plastid-lacking lineages such as *Go. avonlea* will go up or down as databases become more inclusive.

**Fig. 4:**
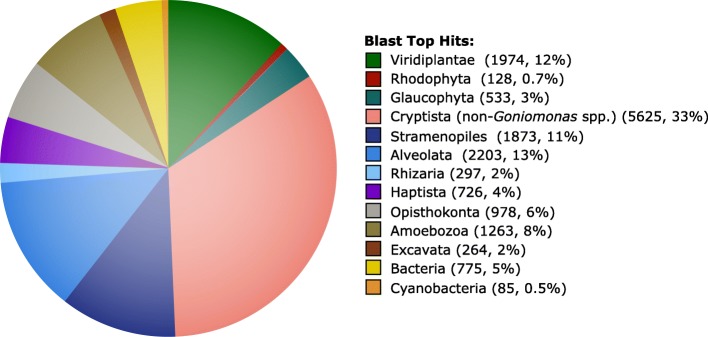
Taxonomic distribution of top blast hits for *Goniomonas avonlea* proteins. The top blast hit was defined as the most significant homolog to *Go. avonlea* (i.e., lowest E-value with a cutoff of 1e^−10^) excluding any other *Goniomonas* sequence

Considering the *Go. avonlea* predicted proteome as a whole, a top blast hit analysis revealed an expected affinity to other Cryptista ~ 33% of the time, with the next most common top hits being to Alveolata (~ 13%), Viridiplantae (~ 12%), and Stramenopiles (~ 11%) (Fig. 4). Notably, the number of instances in which an amoebozoan protein was the most similar sequence (1263 proteins, 8%) was considerably greater than those where a red algal homolog was most similar (128, 0.7%), suggesting again that the red algal signal in the *Go. avonlea* genome is minimal and not the result of EGT. In the case of the phototroph *Gu. theta*, a ~ 4.4 times enrichment in red algal signal relative to *Go. avonlea* was seen in a top blast hit analysis and a ~ 4.5 times greater enrichment in terms of archaeplastidal signal (compared to an amoebozoan control and adjusted for relative database representation to minimize database composition bias [77]). There is thus no indication of a red algal signal above background noise in the *Go. avonlea* genome on the basis of top blast hits.

### The position of Cryptista on the eukaryotic tree of life

**Fig. 5:**
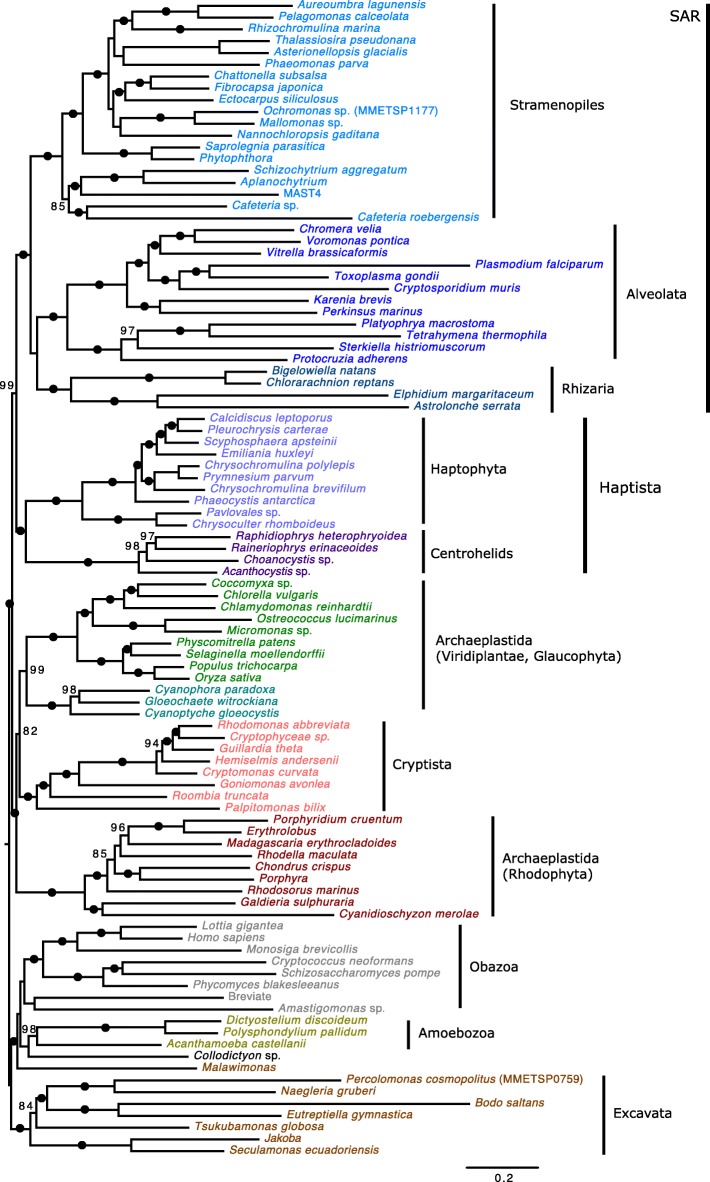
Phylogenomic analysis of the eukaryotic tree of life. Tree shown is a maximum likelihood (ML) phylogeny of a 250 marker gene/protein dataset as in Burki et al. [14] that includes new transcriptome data from *Go. avonlea*. The phylogeny is based on a concatenated marker gene alignment of 71,151 unambiguously aligned sites across 98 OTUs inferred under the model LG + C60 + F + PMSF with 100 standard bootstrap replicates. The tree shown is midpoint rooted. Black dots indicate maximal support for a particular node. When not maximal, only bootstrap support values > 70% are shown. The scale bar shows an inferred 0.2 substitutions per site

The Cryptista comprises a diverse collection of heterotrophic and photosynthetic lineages, one that has only recently been recognized as a monophyletic entity [10, 78]. Not surprisingly, Cryptista has been difficult to place in the eukaryotic tree of life; some or all of its members have been shown to branch sister to Haptophyta (e.g., [8]) or, alternatively, sister to Archaeplastida (e.g., [13]). Phylogenies inferred here based on a modified Burki et al. [68] dataset (98 OTUs, 250 marker genes) recovered identical relationships to those inferred by Burki et al. [14]; however, we were able to evaluate branch support using standard bootstrapping under the complex model LG + C60 + F + PMSF (Fig. 5) [69]. In Burki et al. [14], Bayesian analyses on the original dataset did not result in global convergence, an issue that is common when analyzing such large phylogenomic datasets (e.g., [13]). Nevertheless, these authors considered the tree topology resulting from the non-converged Bayesian analysis and found only minor differences with regard to the position of the Cryptista to Archaeplastida. Due to the large size of our dataset, and in light of the observations of Burki et al. [14], we did not attempt Bayesian analyses; we instead focused on the ML analysis whose tree topology could be statistically evaluated using standard (i.e., nonparametric) bootstrapping. With the exception of Archaeplastida, and the Excavata (whose monophyly is still debated; e.g., see [79]), the monophyly of eukaryotic supergroups (including SAR) was recovered with maximum support, and Haptista (i.e., haptophytes + centrohelids) branched with nearly maximum support as sister to SAR (99% standard bootstrap support). The monophyly of Archaeplastida was disrupted by the positioning of Cryptista, which was found to branch with Archaeplastida with maximum support; more specifically, Cryptista branched with a standard bootstrap value of 82% as sister to Viridiplantae and Glaucophyta (99% standard bootstrap support) to the exclusion of Rhodophyta.

Removing single genes in specific OTUs determined to be discordant via PhyloMCOA [71] did not change the tree topology, but rather significantly increased the support of Cryptista branching internal to Archaeplastida (90% standard bootstrap support), suggesting that the observed relationship is not caused by a few genes in Archaeplastida and Cryptista that overwhelm the dataset with non-phylogenetic signal. The removal of Cryptophyceae from the dataset also resulted in no change in tree topology, recovering non-photosynthetic Cryptista as sister to Viridiplantae and Glaucophyta to the exclusion of Rhodophyta with 75% standard bootstrap support (not substantially different from Fig. 5), suggesting that this association is not entirely due to the presence of plastid-bearing lineages. It remains possible, however, that instances of EGT have gone undetected within Cryptista due to the close evolutionary relationship of their nuclear genes (either Viridiplantae and Glaucophyta specifically or Archaeplastida as a whole) and the source of their plastid (Rhodophyta), making it extremely difficult to disentangle the source of genes in the nucleus and resolve the exact position of the phylum Cryptista within eukaryotes [80].

**Fig. 6:**
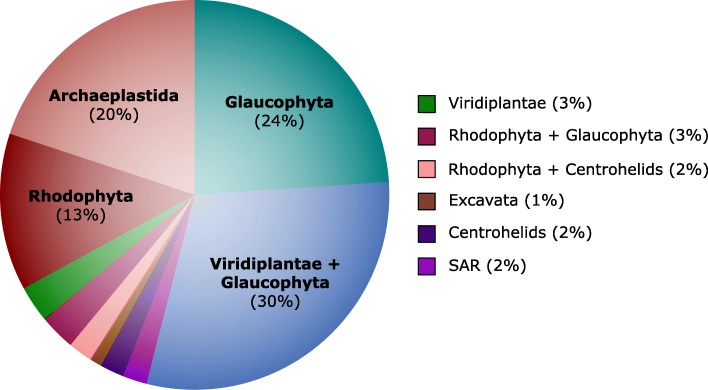
Impact of gene sampling on the phylogenetic position of Cryptista on the tree of eukaryotes. The diagram shows the phylogenetic position of Cryptista within each ML tree inferred using randomly generated subsets of 250 marker genes from the Burki et al. [14] dataset (four gene bins were used; for each iteration, three bins had 46 genes and one bin had 47 genes). Only marker genes for which a homolog was present in *Goniomonas avonlea* and at least one additional Cryptista were included. The distribution shown is based on a total of 100 randomly generated marker gene subset trees

Further investigation into the position of Cryptista on the eukaryotic tree of life using random subsets of marker genes resulted in Cryptista branching consistently with some combination of one or more Archaeplastida sub-groups (93/100 iterations) (Fig. 6). While Cryptista was most frequently observed as sister to the clade comprising Viridiplantae and Glaucophyta (30%), it was also often recovered as sister to Glaucophyta exclusively (24%), Rhodophyta exclusively (13%), and to a monophyletic Archaeplastida (20%). Interestingly, in stark contrast to the 24% of iterations that resulted in a Cryptista-Glaucophyta-specific relationship, only 3% showed Cryptista branching with Viridiplantae exclusively. This may suggest that Cryptista shares a closer ancestry with Glaucophyta, but it could also simply be the result of similar compositional biases or slow evolutionary rates causing “short branch exclusion” [81]. Notably, a sister relationship between Cryptista and Haptophyta/Haptista was never observed. While the exact position of Cryptista relative to Archaeplastida is uncertain, its association with Archaeplastida appears stable. As discussed above, this relationship makes it difficult to assign “algal genes” as EGTs in Cryptista, and it remains to be determined if they are of endosymbiotic origin or vertical ancestry.

### Metabolic “rewiring” in *Guillardia theta* linked to plastid acquisition

Given that there is no plastid in *Go. avonlea*, we compared the predicted metabolic capacities of *Gu. theta* and *Go. avonlea* with the goal of deducing the metabolic and enzymatic functions gained with the acquisition of a red algal-derived secondary plastid. In our study, four main biochemical pathways/processes are predicted to be plastid-localized in *Gu. theta* and thus obviously related to plastid acquisition: photosynthesis, isoprenoid biosynthesis via the non-mevalonate (MEP/DOXP) pathway, carotenoid biosynthesis, and porphyrin and chlorophyll metabolism (Additional file 2: Figure S4–S8). Several other pathways that may also have been acquired by secondary endosymbiosis but are not obviously plastid-localized in *Gu. theta* are ubiquinone and terpenoid-quinone biosynthesis, as well as thiamine biosynthesis (Additional file 2: Figure S9 & S10). As expected, *Gu. theta* pathways clearly localized to the plastid include those associated with pigment biosynthesis and photosynthesis (carotenoid biosynthesis, chlorophyll and porphyrin biosynthesis (Additional file 2: Figure S7 & S8)). The presence of a thiamine (vitamin B1) pathway (Additional file 2: Figure S10), which does not appear to be plastid-localized, as well as ubiquinone and menaquinone/phylloquinone biosynthesis, which are involved in electron transport, also seems to correlate with secondary plastid acquisition in Cryptophyceae. It should be noted that while menaquinone biosynthesis should take place in the plastid, signal peptides have not been detected on the requisite proteins in *Gu. theta* [18]. We also observed that while a peroxisome-localized primary bile biosynthesis pathway is present in *Go. avonlea*, it is apparently absent in *Gu. theta* (Additional file 2: Figure S11). This suggests either loss of this pathway in *Gu. theta* or later acquisition in *Go. avonlea*.

In *Go. avonlea*, fatty acid biosynthesis is predicted to occur partly in the mitochondrion (FabF and FabB) and partly in the cytosol (FAS1) (Additional file 2: Figure S4, S12, S13); while in *Gu. theta*, it is predicted to be plastid-localized. Interestingly, while the mevalonate pathway in *Go. avonlea* is found in the cytosol (Additional file 2: Figure S4, S6), *Gu. theta* possesses both the mevalonate and MEP/DOXP pathways, which use acetyl-CoA and GA3P (d-glyceraldehyde-3-phosphate) with pyruvate, respectively, to synthesize isoprenoid precursor (Additional file 2: Figure S2 and S6). *Gu. theta* (and perhaps other Cryptophyceae) thus appear to have redundant metabolic capacities with which to synthesize isopentenyl diphosphate (i.e., either the mevalonate or the MEP/DOXP pathway) which may represent the ancestral eukaryotic metabolism or the endosymbiotically derived one, respectively.

### Storage polysaccharides in *Goniomonas avonlea*

Alpha glucans are the most common storage polysaccharides and can be found in different forms (e.g., glycogen and starch). Production of alpha glucans can be assessed by the presence of certain CAZyme families: glycoside hydrolase (GH)13, glycosyltransferase (GT)35 and GT5 for all organisms, and GT3, GH133, and GT8 for eukaryotes in particular [82]. These enzymes are all found encoded in the *Go. avonlea* genome and are very similar to those involved in classical eukaryotic glycogen metabolism (Table 2). Several proteins with GT8 domains can putatively be assigned as glycogenins since their best blast hits are to bona fide glycogenins in other organisms such as *Saccharomyces cerevisiae*, albeit with poor E-values (data not shown). Additionally, a complete metabolic pathway for the production of an alpha glucan storage polysaccharide seems to be present in *Go. avonlea*, as supported by the presence of catabolic enzymes such as GH13 and GH14. Because of the presence of the carbohydrate-binding module 45 (CBM45) coupled to a pfam01326 domain (corresponding to a glucan water dikinase (GWD); see below) (Table 2), *Go. avonlea* could be a starch accumulating organism, even though electronic microscopy has not revealed the presence of starch granules [7].

**Table 2:** CAZy family enzymes in *Goniomonas avonlea* and *Guillardia theta* (those predicted to be secreted are in parentheses)

CAZy families	*Go. avonlea*	*Gu. theta*
GH2	5 (4)	4 (0)
GH3	10 (7)	0
GH5	12 (8)	4 (1)
GH9	0	1 (0)
GH13	13 (3)	5 (4)
GH14	2 (1)	3 (1)
GH15	1 (1)	0
GH16	4 (3)	0
GH17	1 (1)	0
GH18	3 (3)	0
GH20	16 (8)	4 (1)
GH22	1 (1)	0
GH24	1 (1)	0
GH25	3 (2)	0
GH27	11 (9)	2 (1)
GH28	5 (4)	0
GH29	5 (4)	2 (1)
GH30	2 (2)	0
GH31	7 (4)	3 (1)
GH32	1 (1)	0
GH33	3 (2)	0
GH35	1 (1)	1 (1)
GH36	0	7 (2)
GH37	1 (0)	0
GH38	9 (6)	1 (0)
GH39	3 (3)	0
GH43	7 (7)	0
GH45	1 (1)	0
GH47	10 (3)	5 (1)
GH50	1 (1)	0
GH51	1 (0)	0
GH54	1 (1)	0
GH55	2 (1)	0
GH56	2 (2)	0
GH63	4 (0)	0
GH65	3 (2)	0
GH76	1 (0)	0
GH77	1 (0)	5 (2)
GH78	6 (3)	0
GH79	4 (3)	1 (1)
GH89	3 (3)	1 (0)
GH92	1 (0)	0
GH95	0	1 (0)
GH99	3 (0)	5 (1)
GH110	1 (1)	0
GH113	1 (0)	0
GH115	1 (0)	0
GH116	2 (2)	1 (0)
GH128	2 (1)	0
GH130	3 (1)	1 (0)
GH133	2 (0)	0
ᅟ	ᅟ	ᅟ
CBM13	1 (1)	1 (0)
CBM20	10 (0)	17 (8)
CBM32	1 (0)	3 (2)
CBM45	1 (0)	0
CBM47	4 (3)	3 (3)
CBM48	9 (0)	6 (2)
ᅟ	ᅟ	ᅟ
GT1	10 (0)	4 (0)
GT2	19 (0)	22 (0)
GT3	2 (0)	0
GT4	18 (0)	27 (0)
GT5	2 (0)	6 (0)
GT6	3 (0)	3 (0)
GT7	1 (0)	2 (1)
GT8	17 (0)	14 (0)
GT10	11 (0)	8 (0)
GT11	4 (0)	3 (0)
GT13	4 (0)	6 (0)
GT14	0	2 (0)
GT15	5 (0)	5 (0)
GT16	1 (0)	3 (0)
GT17	10 (0)	5 (0)
GT18	1 (0)	1 (0)
GT19	1 (0)	1 (0)
GT20	4 (0)	4 (0)
GT22	6 (0)	3 (0)
GT23	16 (0)	16 (0)
GT24	0	1 (0)
GT25	1 (0)	1 (0)
GT26	1 (0)	0
GT28	1 (0)	5 (0)
GT29	0	3 (0)
GT30	1 (0)	1 (0)
GT31	2 (0)	1 (0)
GT32	5 (0)	9 (0)
GT33	1 (0)	1 (0)
GT34	2 (0)	0
GT35	2 (0)	2 (0)
GT37	3 (0)	1 (0)
GT39	1 (0)	1 (0)
GT41	88 (0)	60 (8)
GT47	2 (0)	1 (0)
GT48	1 (0)	0
GT49	7 (0)	19 (0)
GT50	1 (0)	1 (0)
GT54	1 (0)	1 (0)
GT57	3 (0)	2 (0)
GT58	5 (0)	1 (0)
GT59	1 (0)	1 (0)
GT60	1 (0)	1 (0)
GT61	5 (0)	4 (0)
GT64	0	2 (1)
GT66	7 (0)	3 (0)
GT68	0	1 (0)
GT69	0	1 (0)
GT71	1 (0)	1 (0)
GT74	2 (0)	2 (0)
GT75	1 (0)	1 (0)
GT76	1 (0)	1 (0)
GT77	1 (0)	3 (0)
GT90	5 (0)	2 (0)
GT96	8 (0)	2 (0)
ᅟ	ᅟ	ᅟ
CBM50	1 (0)	4 (2)
CBM73	2 (0)	0

*Abbreviations*: *GH* glycoside hydrolase, *GT* glycosyltransferase, *CBM* carbohydrate-binding module

In addition to genes associated with alpha glucan metabolism, the *Go. avonlea* genome encodes putative beta glucan-specific proteins, i.e., enzymes falling in the GT2 and GT48 families (Table 2). These enzymes are implicated in either the production of cellulose in the cell wall or the synthesis of beta storage polysaccharides [83]. No genes for GH9 enzymes were found in the *Go. avonlea* genome, consistent with the fact that cellulose has not been observed in goniomonads [76]. Even if we cannot exclude the possibility of the presence of glucan in the periplast component of cryptomonads, we suggest that the presence of GT2 and GT48 family enzymes could be related to the synthesis of beta storage polysaccharides. The catabolism of beta polysaccharides in *Go. avonlea* could be performed by GH16 family enzymes, laminarinase in particular. However, the laminarinase-like enzymes appear to be secreted, suggesting they are involved in the degradation of exogenous rather than endogenous polysaccharides (Table 2).

### *Goniomonas avonlea* appears capable of digesting both bacteria and eukaryotes

Many heterotrophic eukaryotes ingest bacteria by phagocytosis and *Go. avonlea* is no exception. The CAZy database includes glycoside hydrolases (GHs) clustered into 136 families, and our analysis of carbohydrate-active enzymes (CAZymes) in *Go. avonlea* provides insight into what its prey might be. The *Go. avonlea* genome contains genes for three families of signal peptide-containing lysozymes (GH22, GH24, and GH25) (Table 2) that are likely associated with bacterial phagocytosis. The GH2 family in *Go. avonlea* also includes several enzymes with secretion signals (Table 2). Interestingly, the presence of several GH enzymes suggests that phagocytosis in *Go. avonlea* may also involve eukaryotic prey, specifically algae: these are proteins belonging to the GH45, GH5, and GH3 families, which are putative cellulases, agarases (GH50), and putative hemicellulases (GH43 and GH54; Table 2). Although the cellulases have signal peptides, suggesting that they are involved in the degradation of algal cellulose, it should be noted that cellulose is also found in some bacteria. More intriguing is the identification of genes for signal peptide-containing agarases (GH50) in the *Go. avonlea* nuclear genome, since agar is found only in red algae [84] (Table 2). This suggests that *Go. avonlea* could feed on red algae by phagocytosis, although agarase is also known to degrade alginate, which is found in some bacterial biofilms. The presence of putative secreted hemicellulases in *Go. avonlea* is also consistent with the hypothesis that *Go. avonlea* preys on algae. Several amylases (GH13) and two beta-amylases (GH14) were found to have signal peptides, and may therefore be involved in the degradation of storage polysaccharides from organisms taken up by phagocytosis (Table 2).

While plastid-bearing photosynthetic organisms fix carbon through the Calvin cycle and transform it into sugars for various purposes (most notably, energy), heterotrophic organisms need to acquire sugar from their environment. Thus, photosynthetic organisms typically possess fewer GHs than heterotrophic organisms. This general pattern holds when the heterotroph *Go. avonlea* is compared to the phototroph *Gu. theta. Go. avonlea* possesses 183 GHs (111 of which are predicted to be secreted), compared to only 57 in *Gu. theta* (Table 2). *Gu. theta* also appears to lack certain GH families that are typically absent in autotrophs. Nevertheless, 18 of the 57 GHs in *Gu. theta* are predicted to be secreted, consistent with the gene-based model that predicts *Gu. theta* to be mixotrophic [85], as has been suggested for several other Cryptophyceae [86–88]. Another interesting observation is the co-occurrence of certain GH families in *Gu. theta* and *Go. avonlea*, most notably GH116. However, whereas *Go. avonlea* is predicted to secrete these enzymes in order to obtain exogenous polysaccharides, *Gu. theta* presumably uses them to digest its own endogenous polysaccharides (Table 2). Moreover, while *Gu. theta* does not have more GTs than *Go. avonlea*, some classes that are only present in *Gu. theta* (GT14, GT29) are involved in protein glycosylation (Table 2).

### Global CAZome analysis

**Fig. 7:**
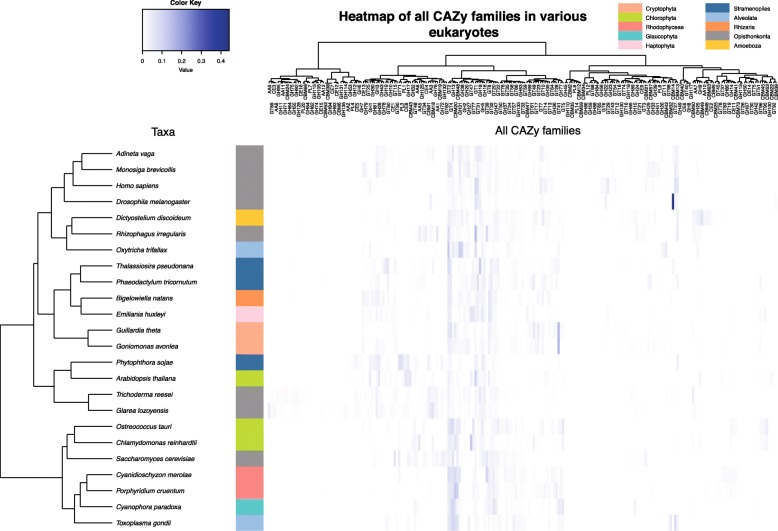
Carbohydrate-active enzyme (CAZyme) families in the heterotroph *Goniomonas avonlea*, the phototroph *Guillardia theta*, and other eukaryotes. Diagram shows a heatmap of CAZyme prevalence in each taxon (abundance within a particular CAZy family divided by the whole number of CAZy families predicted from the genome); the white to blue color scheme indicates low to high prevalence, respectively. Dendogram shows the relative proximity of taxa, or of the co-occurrence of CAZyme families on the left. We observed a close relationship between *Gu*. *theta* and *Go*. *avonlea* (salmon), and that Cryptophyta have a set of CAZyme families similar to that seen in other secondarily photosynthetic algae

In order to better understand the biology of *Go. avonlea* relative to *Gu. theta* and vice versa, we performed a global analysis of the CAZomes of both organisms and compared them to those of other eukaryotes. On the basis of similarity clustering (Additional file 2: Figure S14), we observed a close relationship between the GT families of *Gu*. *theta* and *Go*. *avonlea*. However, when the GH profiles are compared (Additional file 2: Figure S15), *Go*. *avonlea* is more similar to the rotifer *Adineta vaga*, an organism known to be able to degrade cellulose [89], and *Gu. theta* is closer to metazoans. When the CAZome as a whole is analyzed, i.e., all of the carbohydrate-active enzymes predicted for each organism, the *Go*. *avonlea* profile is most similar to that of *Gu*. *theta* (Fig. 7) and, together, these two cryptomonads are generally similar to other organisms with secondarily derived plastids. To determine whether the link between the CAZome profiles of *Go. avonlea* and plastid-bearing organisms is simply due to the presence of *Gu*. *theta*, we removed the latter organism and repeated the clustering analysis (Additional file 2: Figure S16). Even without *Gu. theta*, a specific relationship between the CAZomes of *Go*. *avonlea* and diverse algae (stramenopiles, Haptophyta, Rhizaria) is observed, suggesting that the carbohydrate-active enzyme profile of *Go*. *avonlea* is broadly similar to some algae (Additional file 2: Figure S16). In contrast, when *Go*. *avonlea* is removed from the analysis, *Gu*. *theta* was found to be closest to primary plastid-bearing algae, particularly Chlorophyta and Prasinophyta (Additional file 2: Figure S17). At the present time, the broader significance of these patterns is far from clear, but clearly the CAZomes—and carbohydrate metabolisms—of *Gu*. *theta* and *Go. avonlea* are similar in some ways and different in others.

### From heterotroph to phototroph: the complexity of cryptomonads

Subsequent to the evolution of primary plastids in Archaeplastida, a wide range of eukaryotes acquired photosynthesis secondarily via the engulfment of a red or green algal endosymbiont [2, 4]. Despite more than a decade of cell biological, biochemical, phylogenetic, and phylogenomic investigation, it is still not clear how many times this occurred during the evolution of eukaryotes [6, 90, 91]. On the eukaryotic tree of life, secondary plastid-bearing organisms are scattered amongst plastid-lacking ones, and whether plastid gain or plastid loss has been the dominant mode of organelle evolution has proven difficult to discern.

In the case of red algal-derived plastids, recent evidence strongly suggests a single evolutionary origin of the plastid protein import machinery operating in cryptophytes, haptophytes, photosynthetic stramenopiles, and many alveolates: these organisms all use a host-derived multi-protein complex called SELMA (symbiont-specific endoplasmic reticulum-associated degradation-like machinery) for protein translocation across the second outermost plastid membrane of their four membrane-bound plastids [92–94] (the three membrane-bound plastids of dinoflagellates are an exception). This could mean that the plastids in each of these lineages stem from a single, ancient secondary endosymbiosis involving a red alga and a heterotrophic host (the so-called “chromalveolate” hypothesis [91]), followed by extensive plastid loss in their heterotrophic relatives. Alternatively, one or more cryptic tertiary endosymbioses could have spread the original secondary red plastid (and the genes for SELMA proteins) across the eukaryotic tree (see, e.g., [80, 95, 96]). Distinguishing between these two scenarios is fraught with challenges, not least of which is the fact that the deep structure of the eukaryotic tree of life continues to evolve (see above; Fig. 5) and the biology of many heterotrophic protist lineages remains poorly described.

Using genomic and transcriptomic data, we have explored the metabolic capacities of *Go. avonlea*—the first member of the heterotrophic Goniomonadea to have its genome sequenced—and compared them to those of the model plastid-bearing cryptophyte *Gu. theta*. We have shown that in *Gu. theta*, endosymbiosis led to the gain of metabolic pathways/processes presumably already present in the host, such as fatty acid biosynthesis, as well as de novo acquisition of photosynthesis and the MEP/DOXP pathway (Additional file 2: Figure S5 and S6). Despite its apparent redundancy with the cytosolic mevalonate (MVA) pathway, the plastid-localized MEP/DOXP pathway in *Gu. theta* and other cryptophytes is presumably “useful” given that its end product feeds into other metabolic pathways. For example, the MEP/DOXP pathway generates phytyl-PP, which is used to produce several important components for photosynthesis such as phylloquinone, tocopherol, and chlorophyll [97–99]. Some organisms use the plastid MEP/DOXP pathway instead of the cytosolic MVA pathway, e.g., chlorophyte algae and certain dinoflagellates [100]. In *Gu. theta*, the benefits of photosynthesis presumably offset the costs associated with partial or complete metabolic redundancies. Nevertheless, *Gu. theta* does appear to have lost certain metabolic capacities that were present in its heterotrophic ancestors, such as primary bile biosynthesis (Additional file 2: Figure S4 and S11).

Glycosyl transferases (GTs) catalyze the transfer of sugars from donor to acceptor molecules, thereby creating glycosidic bonds. The evolution of photosynthesis is generally associated with the acquisition of new GT families and this is indeed what seems to have happened in the cryptophytes. Interestingly, however, we did not observe a significant increase in the total number of genes for GT enzymes in *Gu. theta* relative to *Go. avonlea*, and in fact, some GTs are present in *Go. avonlea* but not in *Gu. theta*; some GTs (GT3, GT48, GT34) appear to have been lost in *Gu. theta* and others replaced (e.g., GT3 has probably been replaced by a GT5 in *Gu. theta*) [82]. In addition, the loss of GT48 seems to correlate with the loss of beta glucan synthesis, as *Gu. theta* appears capable of generating only alpha-glucan polysaccharides, as in the red algal progenitor of its plastid.

**Fig. 8:**
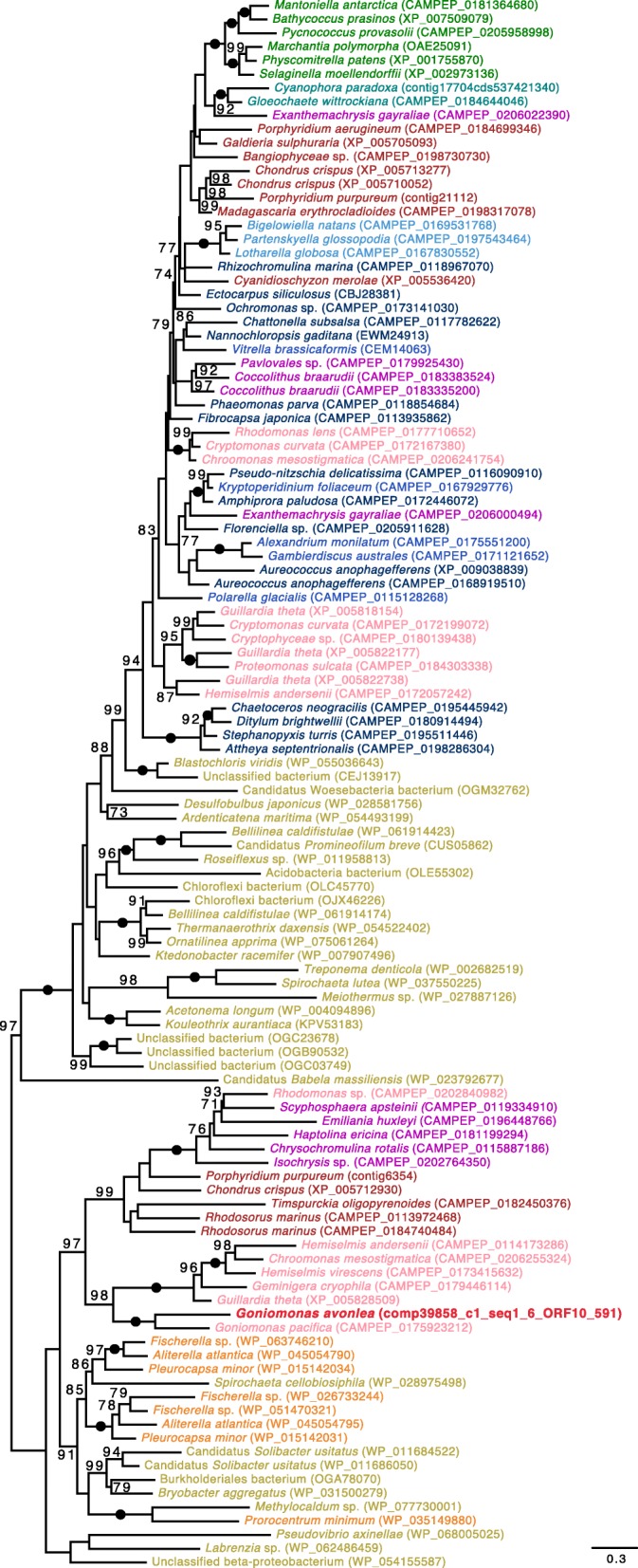
Phylogenetic analysis of glycosyltransferase (GT) 28. The tree shown is a maximum likelihood tree with ultrafast bootstrap values mapped onto the nodes. The tree shown is midpoint rooted. Sequences are colored according to their taxonomic affiliation: Viridiplantae are in green, Glaucophyta are in turquoise, Rhodophyta are in dark red, Cyanobacteria are orange and other Bacteria are in gold, Cryptophyta are in pink, *Goniomonas avonlea* is dark red and bolded, Haptophyta are in purple, Stramenopiles are in dark blue, Alveolata are in blue, Rhizaria are in light blue. The GT28 from *Go. avonlea* groups with other cryptomonads and with Rhodophyta. It is noteworthy that GT28 grouping with *Go*. *avonlea* seem to be mitochondrial based on signal targeting prediction while GT28 on the upper part could be targeted to the plastid, based on targeting prediction signal. The scale bar shows the inferred number of amino acid substitutions per site

Most unexpected was the discovery of a sequence of the GT28 family in *Go. avonlea* (Table 2), a category of enzymes known to be involved in the synthesis of cell wall components in bacteria (MurG is a GT28 enzyme acting as a UDP-*N*-acetylglucosamine:lipopolysaccharide *N*-acetylglucosamine transferase) and the synthesis of galactoglycerolipids in plastid-bearing organisms (MGD is a GT28 enzyme synthesizing 1,2-Diacyl-3-beta-d-galactosyl-sn-glycerol). All GT28-containing eukaryotes in the public CAZy database have plastids (http://www.cazy.org/GT28_eukaryota.html), and more recent investigation shows that of the more than 1000 eukaryotes that have had their CAZomes annotated, only eight have GT28 genes and lack plastids (most of these are fungi; data not shown). The GT28 gene in *Go. avonlea* resides on a contig with six other genes, five of which have a top blast hit to another eukaryote (2 being *Gu*. *theta*), and the sixth is a bacterial-like gene that also has a close homolog in the *Gu. theta* nuclear genome (Additional file 2: Figure S18). In addition, the GT28 gene in *Go*. *avonlea* contains spliceosomal introns, confirming its provenance as a eukaryotic nuclear gene. The exact function of the *Go. avonlea* GT28 enzyme is difficult to predict with confidence. Based on sequence similarity, it could be a 1,2-diacylglycerol 3-beta-galactosyltransferase [EC 2.4.1.46] and is predicted to be targeted to the mitochondrion by TargetP and PredSL; this is also thought to be the case in some non-photosynthetic algae [101]. We speculate that the *Go. avonlea* enzyme might be involved in the synthesis of phosphate-free mitochondrial lipids, which could serve in a phospholipid-to-galactolipid exchange as observed in *Arabidopsis thaliana* mitochondria during phosphate starvation [102]. The origin and function of the GT28 gene in *Go. avonlea* thus appears distinct from the MGD genes of plastid-bearing eukaryotes. Biochemical, physiological, and ideally functional genomic studies need to be undergone to solve this question. Regardless of its precise function, it is noteworthy that in phylogenetic analyses, the GT28 homologs of *Go. avonlea* and *Go. pacifica* branch robustly with cryptophytes (including *Gu. theta*), red algae, and haptophytes (Fig. 8).

**Fig. 9:**
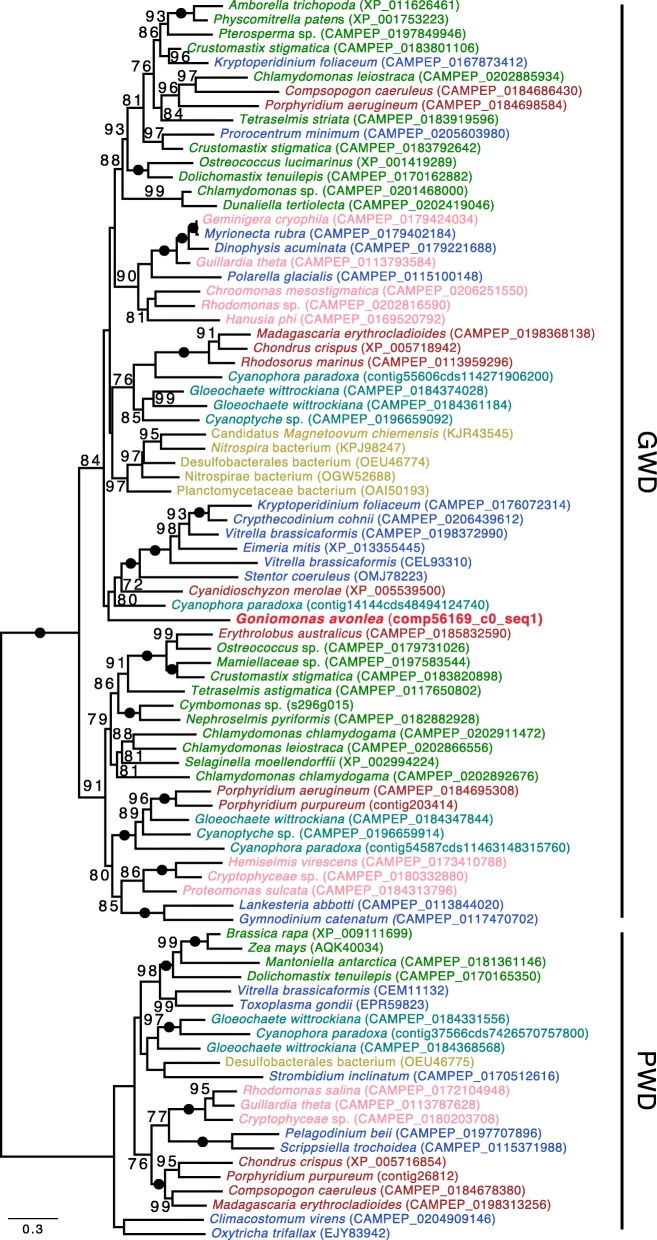
Glucan Water Dikinase (GWD) phylogenetic tree. The tree shown is a maximum likelihood tree with ultrafast bootstrap values mapped onto the nodes. The tree is rooted with the Phosphoglucan dikinase (PWD) sequences. Sequences are colored according to their taxonomic affiliation: Viridiplantae are in green, Glaucophyta are in turquoise, Rhodophyta are in dark red, Cyanobacteria are orange and other Bacteria are in gold, Cryptophyta are in pink, *Goniomonas avonlea* is dark red and bolded, Haptophyta are in purple, Stramenopiles are in dark blue, Alveolata are in blue, Rhizaria are in light blue. Some bacteria (in gold) could have obtained their GWD gene by LGT. The GWD homolog from *Go. avonlea* branches close to its counterpart in Rhodophyta and Glaucophyta; GWDs in Cryptophyta appear more distantly related. The scale bar shows the inferred number of amino acid substitutions per site

Another unexpected carbohydrate-active enzyme in *Go. avonlea* is glucan water dikinase (GWD). The discovery of GWD in a goniomonad is unexpected because it has long been assumed that *Gu. theta* and other cryptophytes acquired starch metabolism as a result of the acquisition of its secondary plastid. GWD has thus far only been found in organisms known to accumulate starch; the enzyme has been proposed to have evolved concomitantly with the primary plastid found in Viridiplantae, Rhodophyta, and Glaucophyta (i.e., Archaeplastida) [103]. Indeed, all eukaryotic starch accumulators either have a plastid or have been proposed to have once had a plastid—such as ciliates [104]—at some point during their evolution [82, 103] (Fig. 9). In *Go. avonlea*, the GWD gene contains introns and resides on a contig with seven other genes, three of which are clearly of eukaryotic provenance (Additional file 2: Figure S19). Given the absence of obvious starch granules in *Go. avonlea*, it is possible that the organism only synthesizes starch at certain stages of its life cycle, as is the case in the apicomplexan *Toxoplasma gondii* [105]. In phylogenetic analyses, the *Go. avonlea* GWD homolog does not branch with cryptophytes, but rather is weakly associated with a clade containing sequences from Rhodophyta, Glaucophyta, and various algae with red algal type plastids. How *Go. avonlea* came to possess and retain its GWD gene is an open question.

## Conclusions

We have sequenced the nuclear genome of *Goniomonas avonlea*, the first of its kind for a plastid-lacking cryptomonad and only the second to be sequenced for all of Cryptista. The *Go. avonlea* genome provides a much-needed first glimpse into the biology of a heterotrophic protist residing on a large, poorly understood branch of the eukaryotic tree. Amongst the 33,470 predicted protein-coding genes in the *Go. avonlea* genome are hundreds of genes for carbohydrate-active enzymes that provide important clues as to what this phagotrophic protist eats in nature—the organism appears capable of digesting bacteria as well as eukaryotes, including algae. We found no convincing phylogenetic evidence to support the notion that *Go. avonlea* evolved from a secondary plastid-bearing ancestor; in terms of abundance, the handful of “algal” genes in the genome do not rise above background “noise.” Nevertheless, this enigmatic protist possesses genes for enzymes such as GT28 and GWD, which are almost invariably found in plastid-bearing organisms. This is interesting for various reasons, not least of which is the fact that analysis of a 250-protein dataset placed cryptophytes, goniomonads (including *Go. avonlea*), and other heterotrophic Cryptista within the primary plastid-bearing Archaeplastida. While *Go. avonlea* serves as an important reference point for studying the metabolic transformation that took place during secondary endosymbiosis in the ancestor of modern-day Cryptophyceae, aspects of biochemistry and molecular biology may be linked to its deep ties with primary plastid-bearing organisms. More genomic data from diverse heterotrophic members of the Cryptista will hopefully allow us to test this hypothesis.

## Additional files

Additional file 1: Flowchart summarizing sub-cellular localization predictions for *Goniomonas avonlea* proteins. (PDF 95 kb)

Additional file 2: **Table S1.** BUSCO analysis of *Goniomonas avonlea*, *Guillardia theta*, and *Dictyostelium discoideum* proteomes. **Figure S1.** Top: mitochondrial genome of *Goniomonas avonlea*. Bottom: Gene presence/absence matrix for Cryptophyta and other eukaryotes. **Figure S2.** KEGG map of the metabolic pathways in *Go. avonlea* compared to plastid/periplastidal pathways in *Gu. theta*. **Figure S3.** KEGG map of metabolic pathways in a putative secondary plastid in *Go. avonlea* compared to plastid/periplastidal pathways in *Gu. theta* and *Arabidopsis*. **Figure S4.** Metabolic maps for *Go. avonlea* and *Gu. theta*. **Figure S5.**–**S13.** KEGG representation of photosynthesis (Figure S5), terpenoid backbone biosynthesis (**Figure S6.**), carotenoid biosynthesis (**Figure S7.**), porphyrin and chlorophyll metabolism (**Figure S8.**), ubiquinone biosynthesis (**Figure S9.**), thiamine metabolism (**Figure S10.**), primary bile acid biosynthesis (**Figure S11.**), cellular metabolism (**Figure S12.**), and fatty acid biosynthesis (**Figure S13.**) in *Go. avonlea*. **Figure S14.** Comparison of GlycosylTransferase (GT) CAZy families in *Go. avonlea* and other eukaryotes. **Figure S15.** Comparison of Glycoside Hydrolase CAZy families in *Go. avonlea* and other eukaryotes. **Figure S16.** Comparison of all CAZy families in *Go. avonlea* and other eukaryotes. **Figure S17.** CAZy prevalence in various eukaryotes. **Figure S18.** Genomic context of GT28 in *Go. avonlea*. **Figure S19.** Genomic context of glucan water dikinase (GWD) in *Go. avonlea*. (PDF 11691 kb)
