# Supplementary material for: Nuclear genome sequence of the plastid-lacking cryptomonad Goniomonas avonlea provides insights into the evolution of secondary plastids
Source: BMC Biol. 2018 Nov 28;16:137. doi: 10.1186/s12915-018-0593-5 (PMC6260743; doi:10.1186/s12915-018-0593-5)

Supplementary Data 1—Flowchart summarizing sub-cellular localization predictions for *Goniomonas avonlea* proteins. Abbreviation: PPC, periplastidial compartment.

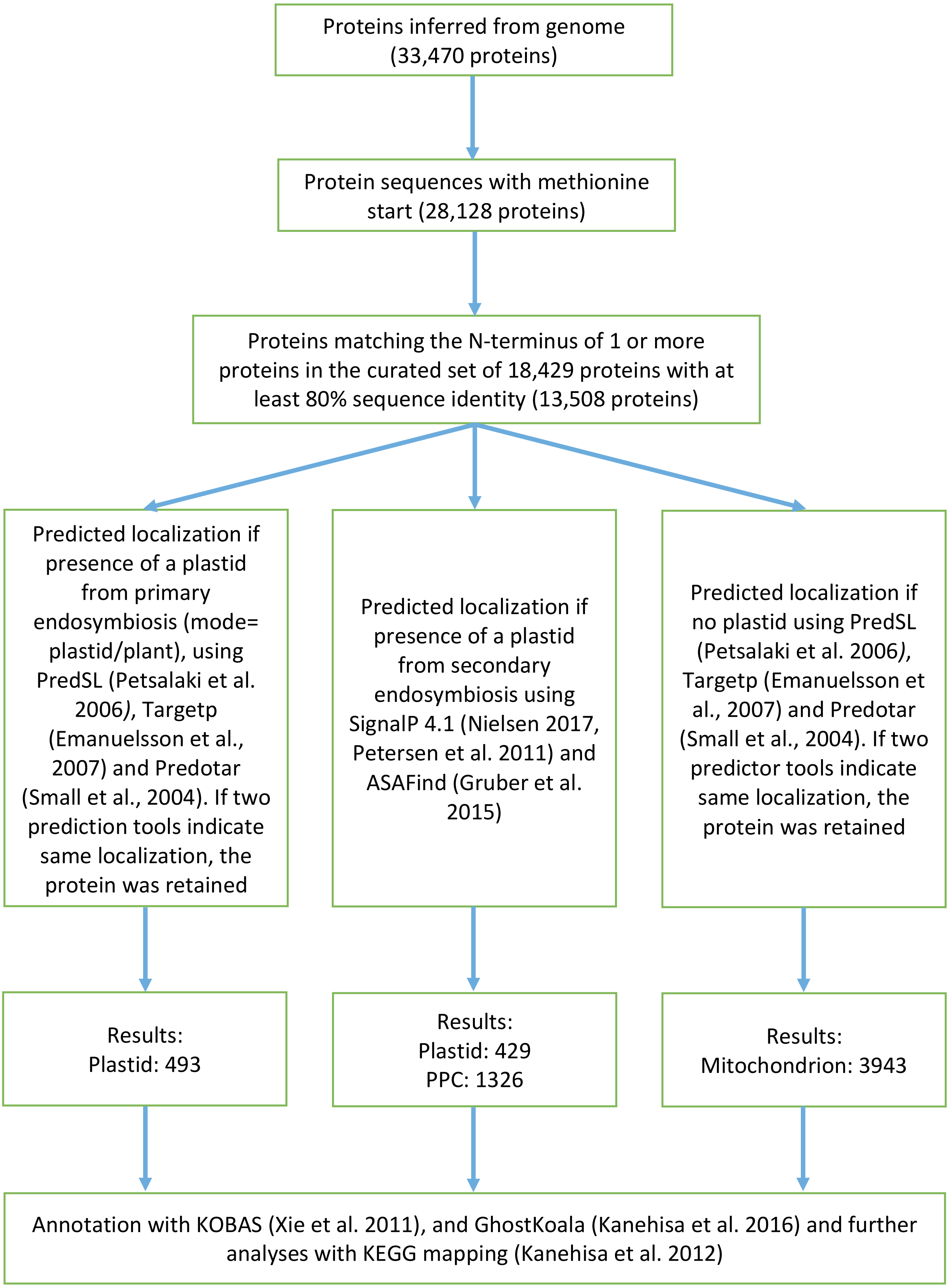

Supplement: Supplementary file 1 — Flowchart summarizing sub-cellular localization predictions for Goniomonas avonlea proteins. (PDF 95 kb) [file 12915_2018_593_MOESM1_ESM.pdf]
