# Supplementary material for: Nuclear genome sequence of the plastid-lacking cryptomonad Goniomonas avonlea provides insights into the evolution of secondary plastids
Source: BMC Biol. 2018 Nov 28;16:137. doi: 10.1186/s12915-018-0593-5 (PMC6260743; doi:10.1186/s12915-018-0593-5)
Supplement: Supplementary file 2 — Table S1. BUSCO analysis of Goniomonas avonlea, Guillardia theta, and Dictyostelium discoideum proteomes. Figure S1. Top: mitochondrial genome of Goniomonas avonlea. Bottom: Gene presence/absence matrix for Cryptophyta and other eukaryotes. Figure S2. KEGG map of the metabolic pathways in Go. avonlea compared to plastid/periplastidal pathways in Gu. theta. Figure S3. KEGG map of metabolic pathways in a putative secondary plastid in Go. avonlea compared to plastid/periplastidal pathways in Gu. theta and Arabidopsis. Figure S4. Metabolic maps for Go. avonlea and Gu. theta. Figure S5.–S13. KEGG representation of photosynthesis (Figure S5), terpenoid backbone biosynthesis (Figure S6.), carotenoid biosynthesis (Figure S7.), porphyrin and chlorophyll metabolism (Figure S8.), ubiquinone biosynthesis (Figure S9.), thiamine metabolism (Figure S10.), primary bile acid biosynthesis (Figure S11.), cellular metabolism (Figure S12.), and fatty acid biosynthesis (Figure S13.) in Go. avonlea. Figure S14. Comparison of GlycosylTransferase (GT) CAZy families in Go. avonlea and other eukaryotes. Figure S15. Comparison of Glycoside Hydrolase CAZy families in Go. avonlea and other eukaryotes. Figure S16. Comparison of all CAZy families in Go. avonlea and other eukaryotes. Figure S17. CAZy prevalence in various eukaryotes. Figure S18. Genomic context of GT28 in Go. avonlea. Figure S19. Genomic context of glucan water dikinase (GWD) in Go. avonlea. (PDF 11691 kb) [file 12915_2018_593_MOESM2_ESM.pdf]

Supplementary data 2, Table S1. BUSCO analysis of the *Goniomonas avonlea*, *Guillardia theta*, and *Dictyostelium discoideum* proteomes

Genome	Complete	Duplicated	Fragmented	Missing
Go. avonlea	69%	19%	20%	9.7%
G. theta	78%	32%	12%	8.8%
D. discoideum	93%	28%	1.8%	5.1%

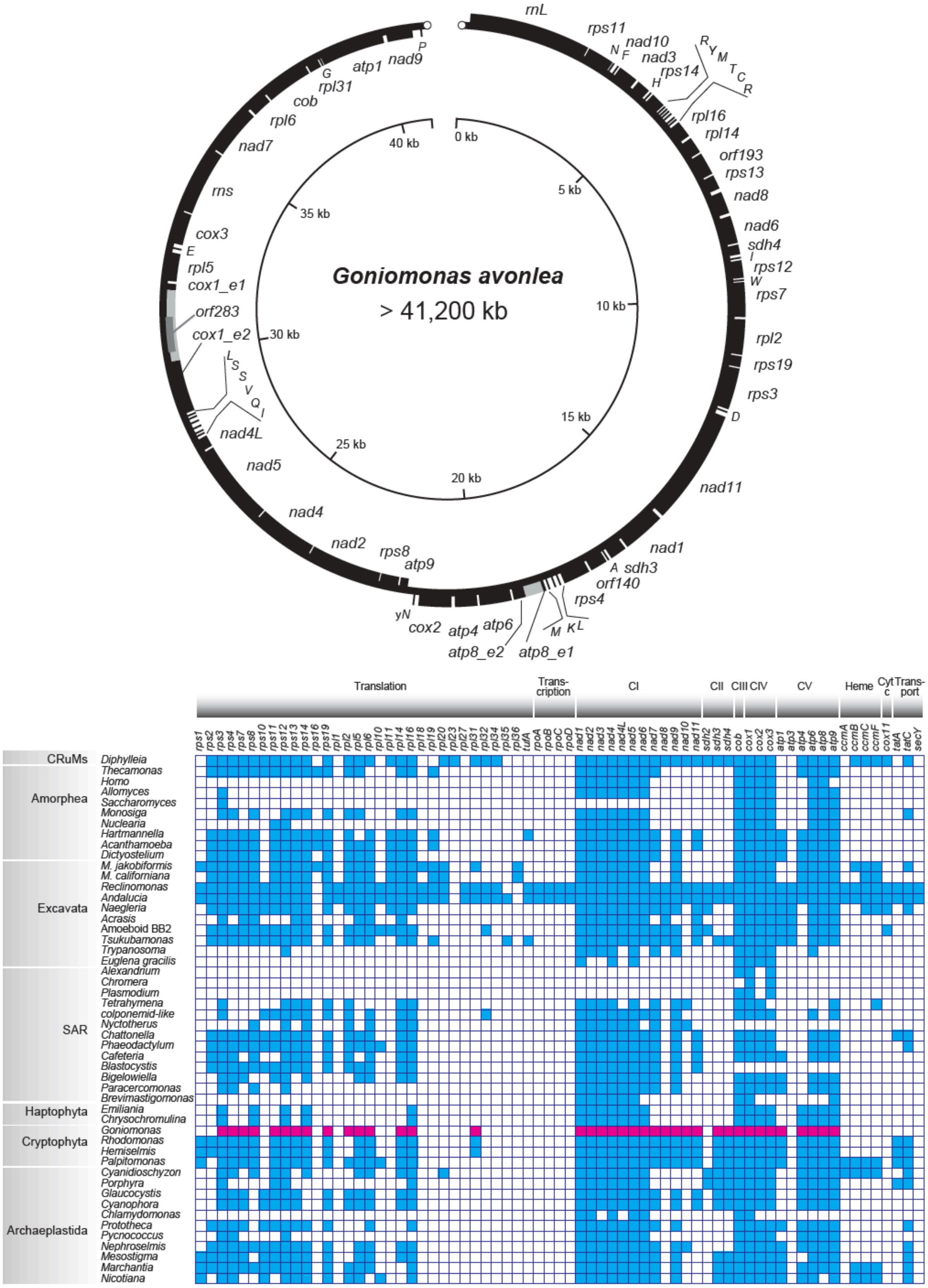

Supplementary data 2, **Figure S1**. Top: mitochondrial genome of *Goniomonas avonlea*. Bottom: Gene presence/absence matrix for Cryptophyta and other eukaryotes. The *Go. avonlea* genes are highlighted pink.

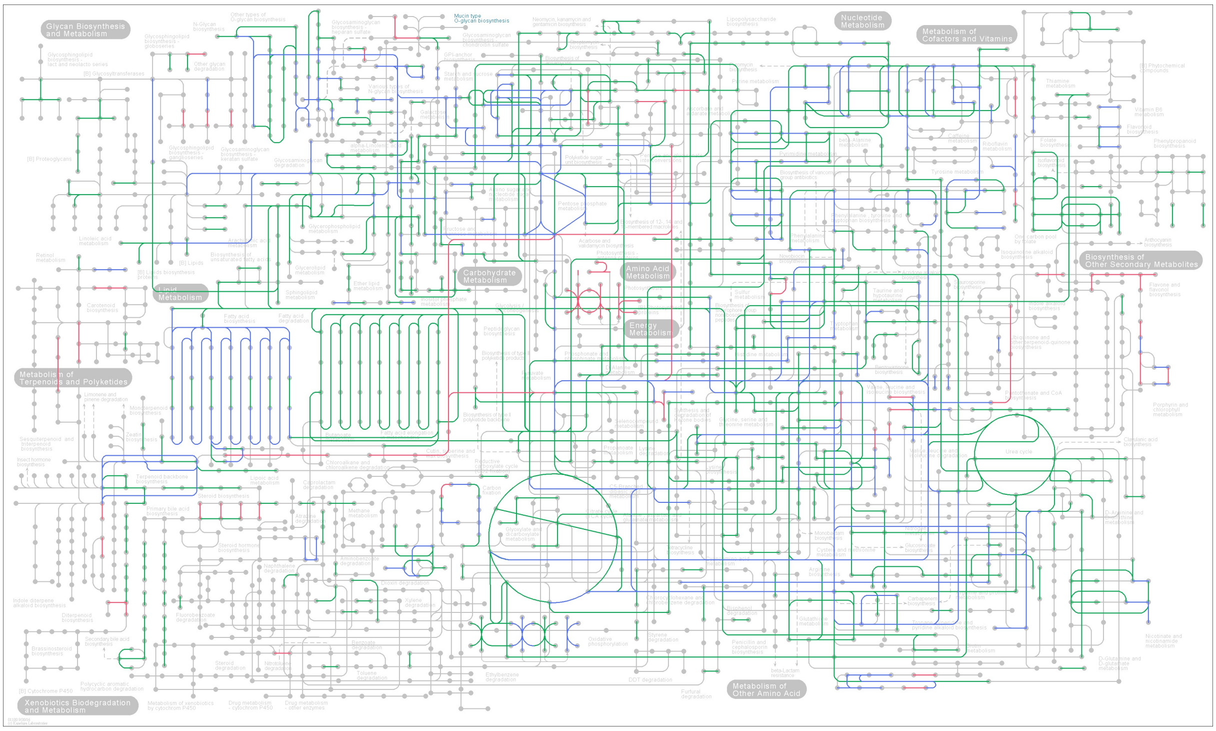

Supplementary data 2, Figure S2: KEGG map of the metabolic pathways in *Go. avonlea* (green) compared to pathways predicted to be in the plastid or periplastidial compartment (PPC) of *Gu. theta* (red). The blue lines indicate pathways found in both PPC or plastid of *Gu. theta* and in *Go. avonlea*. Typical plastid metabolic pathways/processes include the Calvin cycle, photosynthesis pigment biosynthesis (carotenoid and chlorophyll), and menaquinone biosynthesis. None of those metabolisms, however, are found in *Go. avonlea*. This map also shows that despite lack of plastid-associated metabolisms, *Go. avonlea* shares some pathways localized in the plastid and the PPC, such as fatty acid biosynthesis, the first step of the isoprenoid backbone biosynthesis.

A

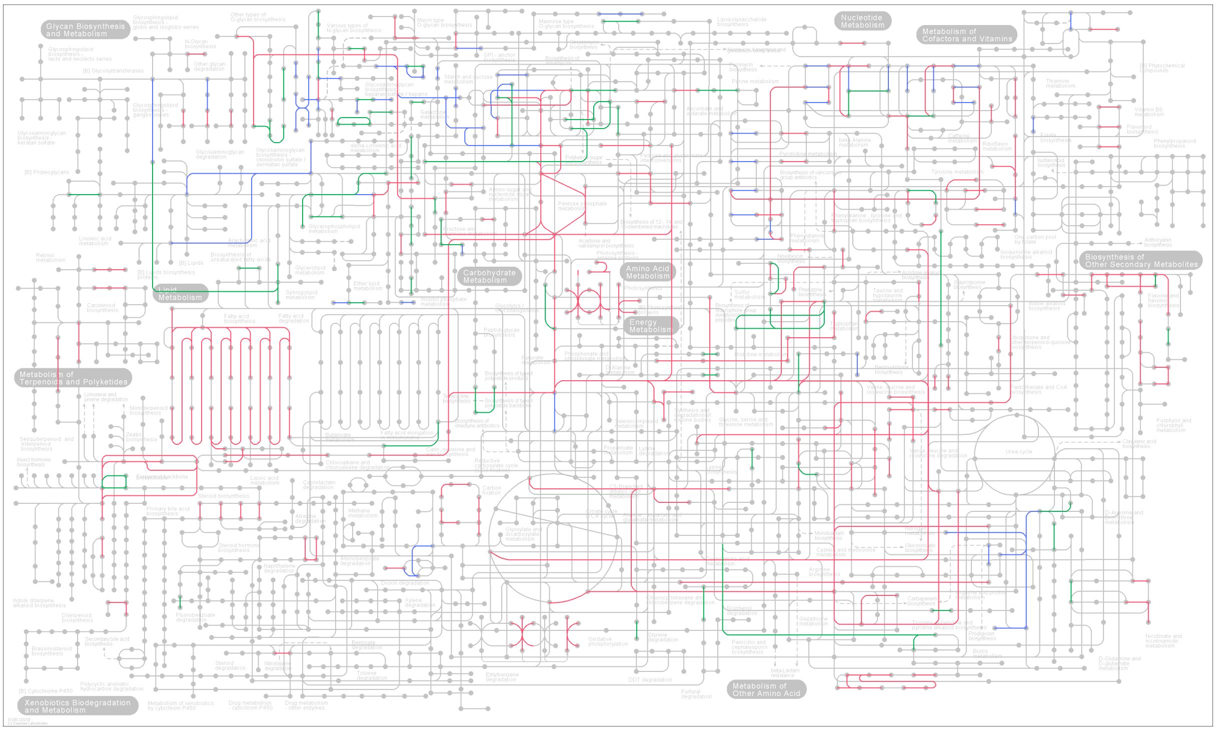

B

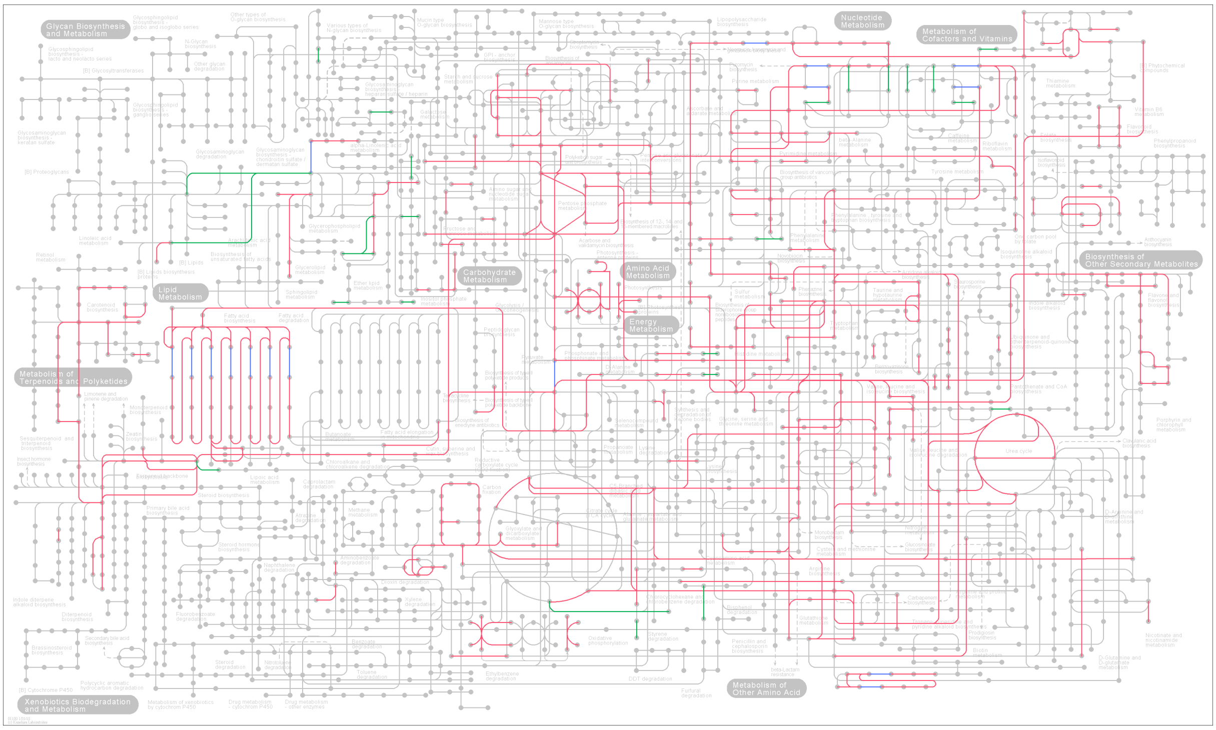

Supplementary data 2, Figure S3: KEGG map of the metabolic pathways in a putative plastid from secondary plastid endosymbiosis in *Go. avonlea* (green) compared to pathways predicted to be in the plastid and PPC of *Gu. theta* (red) (A) or in a putative plastid from primary endosymbiosis in *Go. avonlea* (green) (B), compared to pathways in the *Arabidopsis* plastid (red). Blue lines indicate pathways found in both organisms. We observed mostly red lines meaning that only a few pathways in both *Gu. theta* plastid+PPC and the *Arabidopsis* plastid are predicted to be in *Go. avonlea* in the same subcellular compartments. All things considered, we found no evidence supporting the presence of a plastid of primary or secondary endosymbiotic ancestry in *Go. avonlea*.

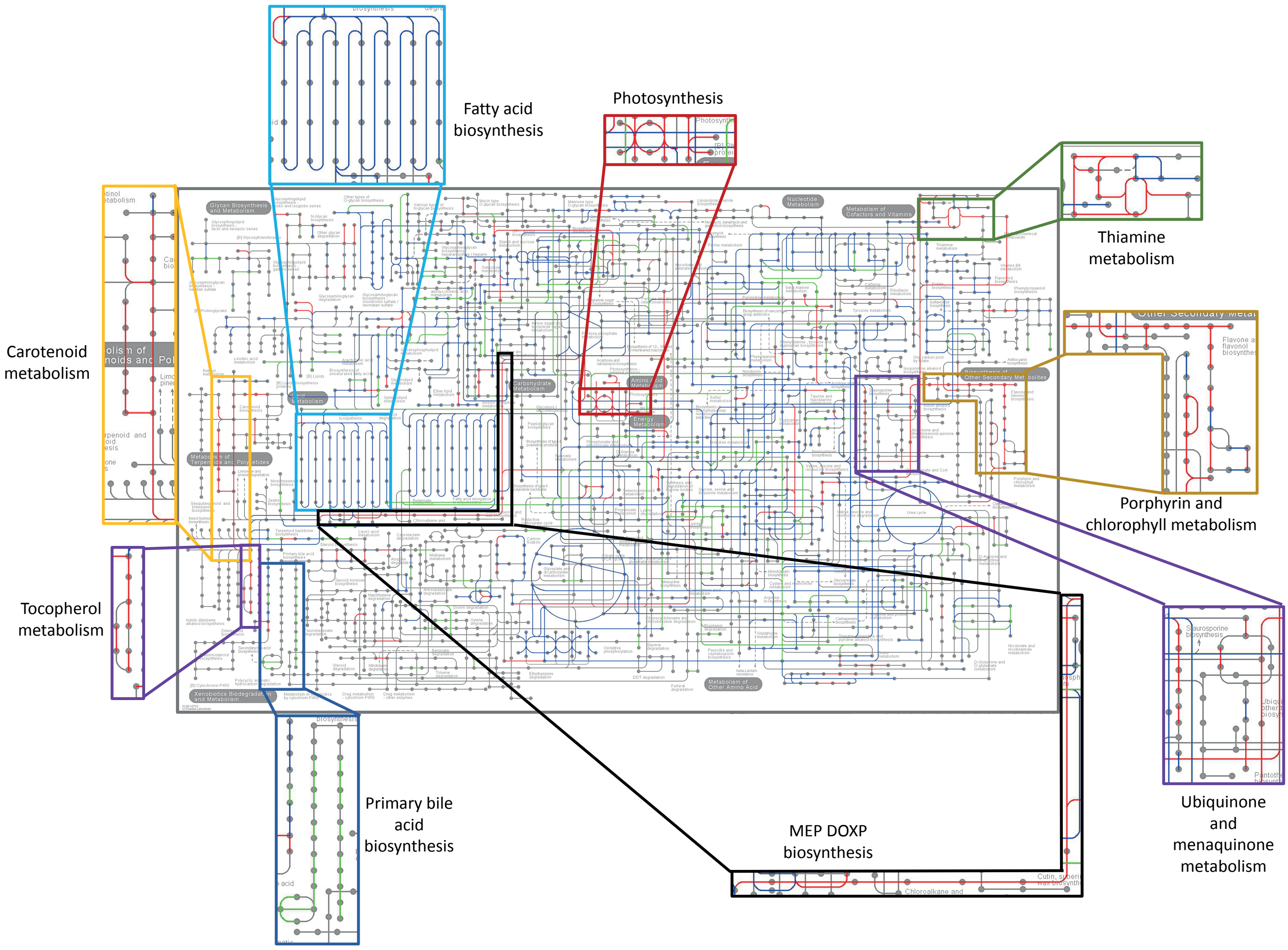

Supplementary data 2, Figure S4: Metabolic maps for *Goniomonas avonlea* and *Guillardia theta*. Nodes represent metabolic compounds and lines represent enzyme-catalyzed biochemical reactions. Metabolic maps were based on KEGG annotations and generated using KEGG Mapper (http://www.genome.jp/kegg/tool/map_pathway.html). Colored lines indicate enzymes/reactions that are predicted to be present in *Goniomonas avonlea* (green), *Guillardia theta* (red), or both organisms (blue). Enlargements are shown for: photosynthesis, MEP/DOXP pathway, carotenoid metabolism, porphyrin and chlorophyll metabolism, the ubiquinone and menaquinone biosynthesis, the tocopherol biosynthesis, thiamine metabolism, primary bile acid biosynthesis, and fatty acid biosynthesis.

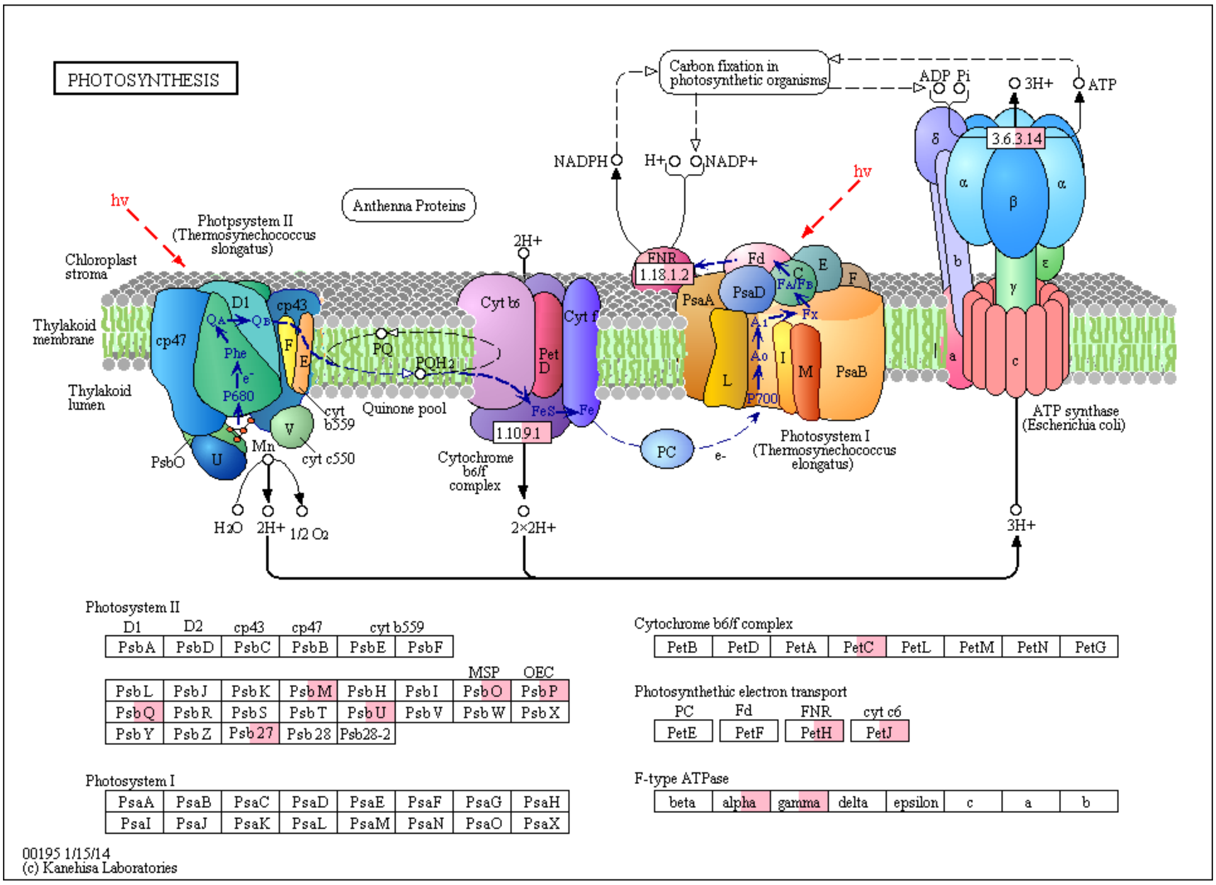

Supplementary data 2, Figure S5: KEGG representation of photosynthesis, with the red in each rectangle indicating the predicted presence of this protein in *Gu. theta*.

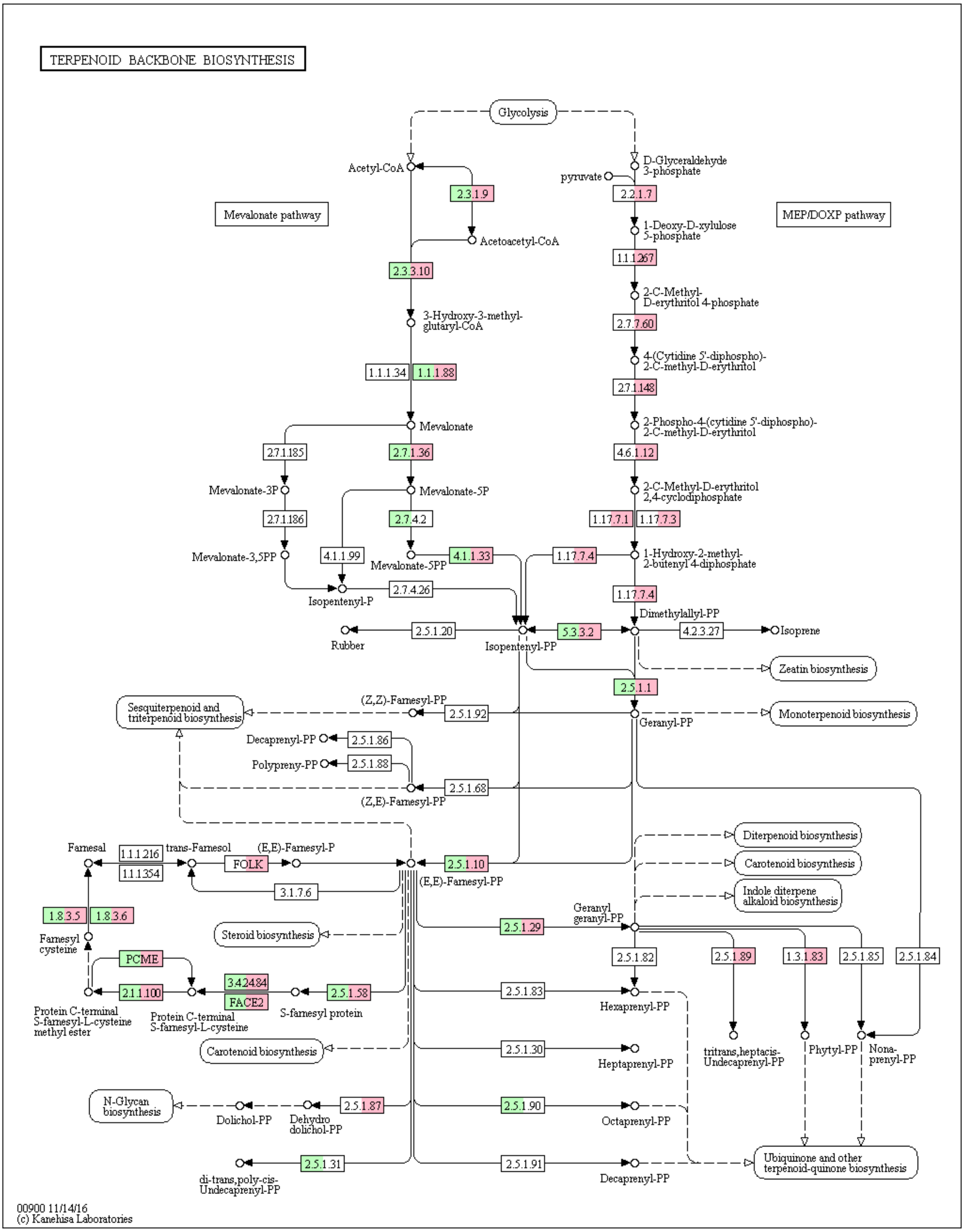

Supplementary data 2, Figure S6: KEGG representation of terpenoid backbone biosynthesis. Proteins present in *Go. avonlea* are indicated by green coloring of the corresponding rectangle while red indicates protein presence in *Gu. theta*. The evidence suggests that synthesis of phytyl-PP, which is used in phylloquinone biosynthesis, and the MEP/DOXP pathway, are only found in *Gu. theta*.

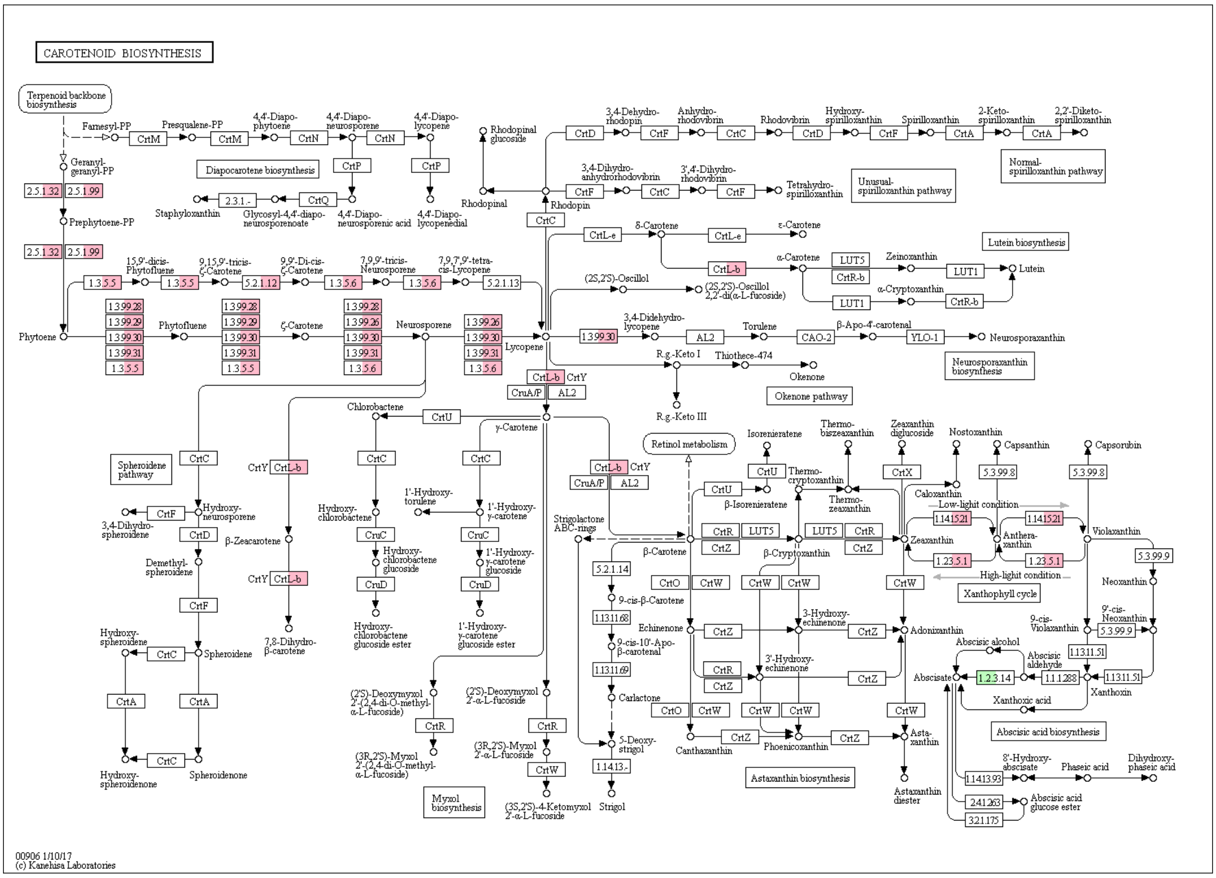

Supplementary data 2, Figure S7: KEGG representation of carotenoid biosynthesis. Proteins present in *Go. avonlea* are indicated in green while red indicates proteins in *Gu. theta*. Carotenoid synthesis appears to be restricted to *Gu. theta*.

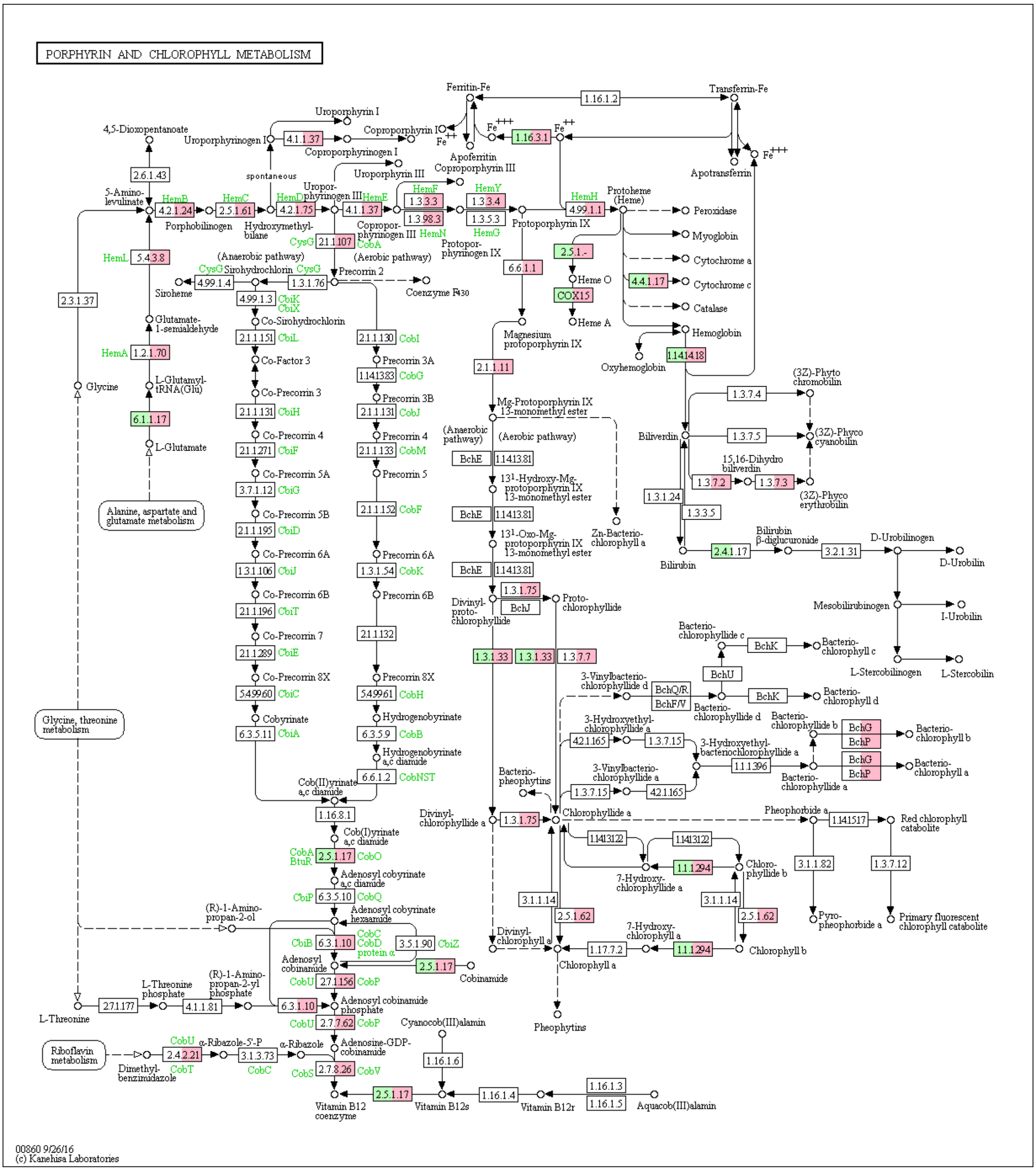

Supplementary data 2, Figure S8: KEGG representation of porphyrin and chlorophyll metabolism. Proteins predicted to be present in *Go. avonlea* are indicated by a green rectangle while red indicates proteins present in *Gu. theta*. The heme pathway and vitamin B12 synthesis both seem to be present only in *Gu. theta*, suggesting that *Go. avonlea* acquires heme and vitamin B12 through phagotrophy.

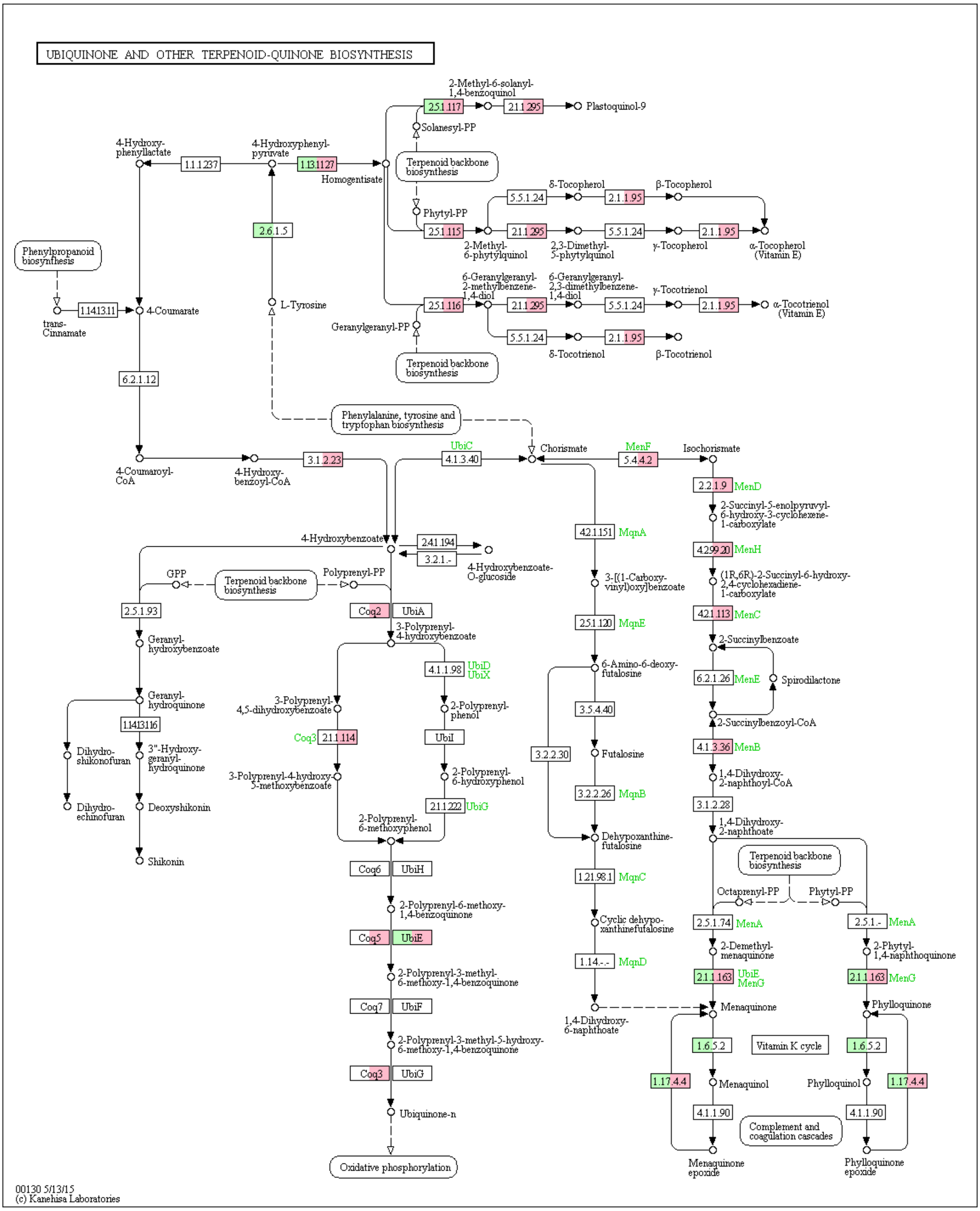

Supplementary data 2, Figure S9: KEGG representation of ubiquinone and other terpenoid-quinone biosynthesis. Proteins present in *Go. avonlea* are indicated by green rectangles while red rectangles indicate proteins present in *Gu. theta*. The ubiquinone pathway, as well as menaquinone / phylloquinone biosynthesis, is only found in *Gu. theta*. Detection of menaquinone / phylloquinone biosynthesis in *Gu. theta* is expected since phylloquinone can transport electrons during photosynthesis. Menaquinone / phylloquinone biosynthesis is probably plastid-localized, despite the fact that our subcellular localization predictions failed to predict the presence of this pathway inside the plastid.

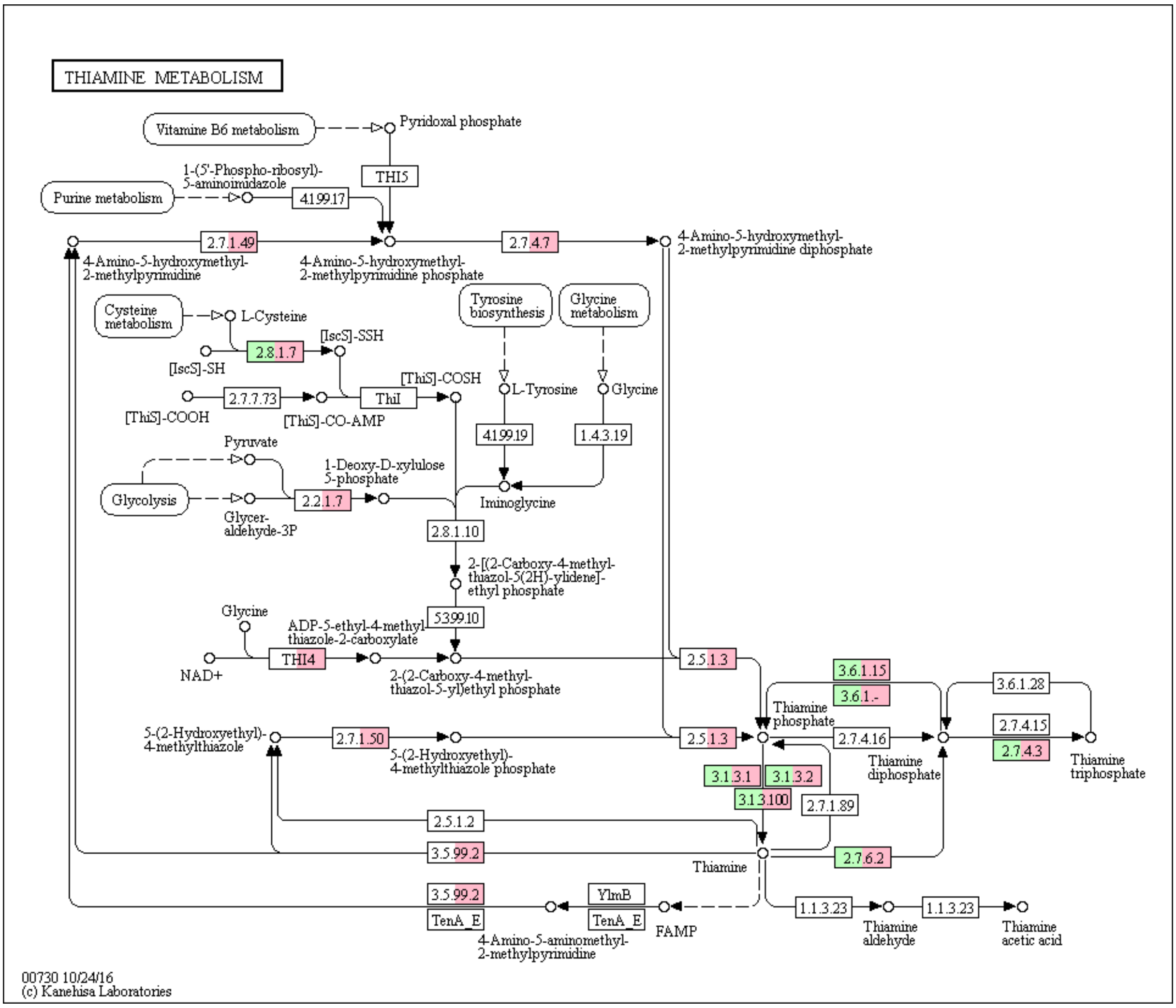

Supplementary data 2, Figure S10: KEGG representation of thiamine metabolism. Proteins present in *Go. avonlea* are indicated by a green box while red indicates proteins in *Gu. theta*.

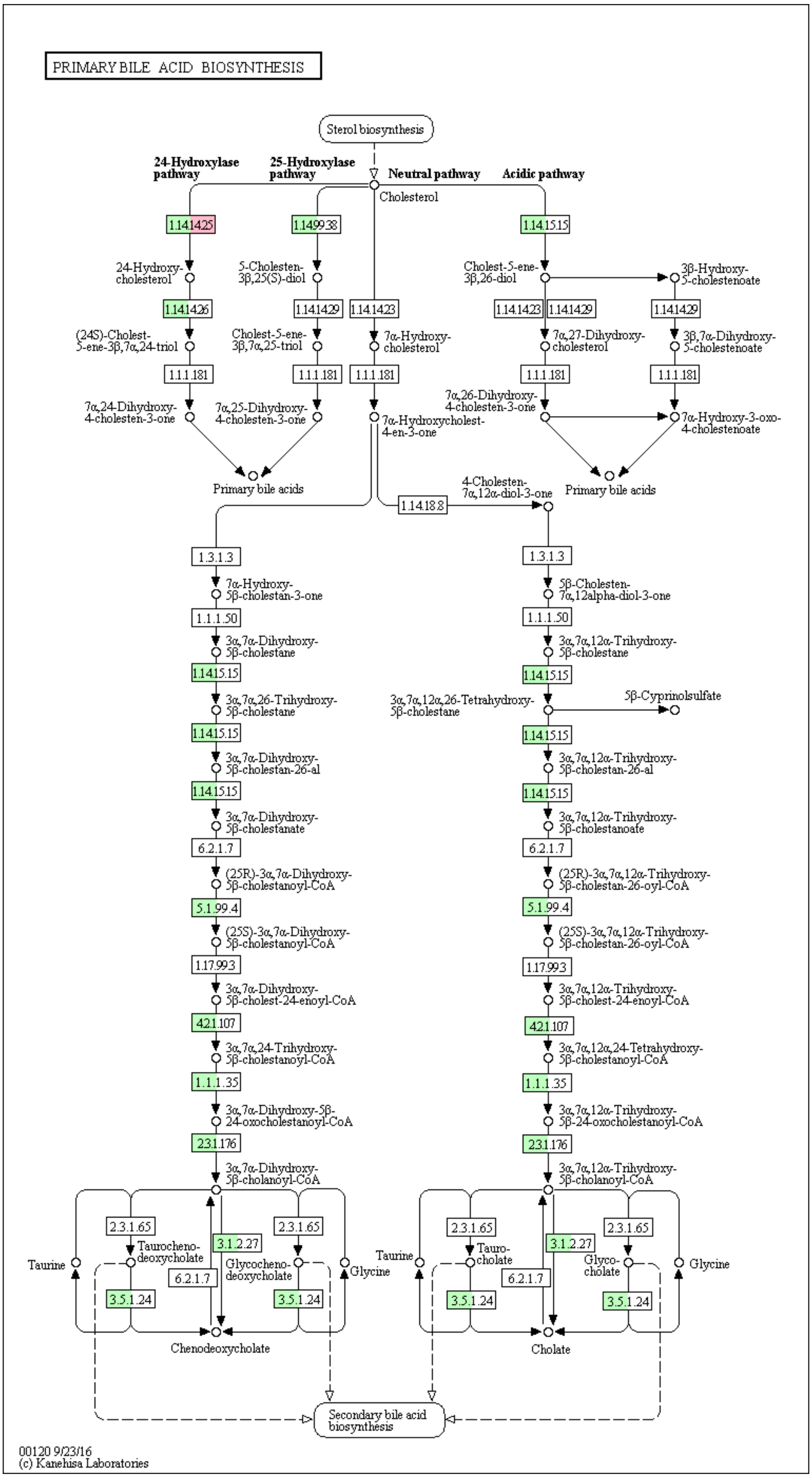

Supplementary data 2, Figure S11: KEGG representation of primary bile acid biosynthesis. Green rectangles indicate the predicted presence of a protein in *Go. avonlea* while red coloring indicates proteins present in *Gu. theta*. Primary bile biosynthesis is only found in *Go. avonlea* and not in *Gu. theta*.

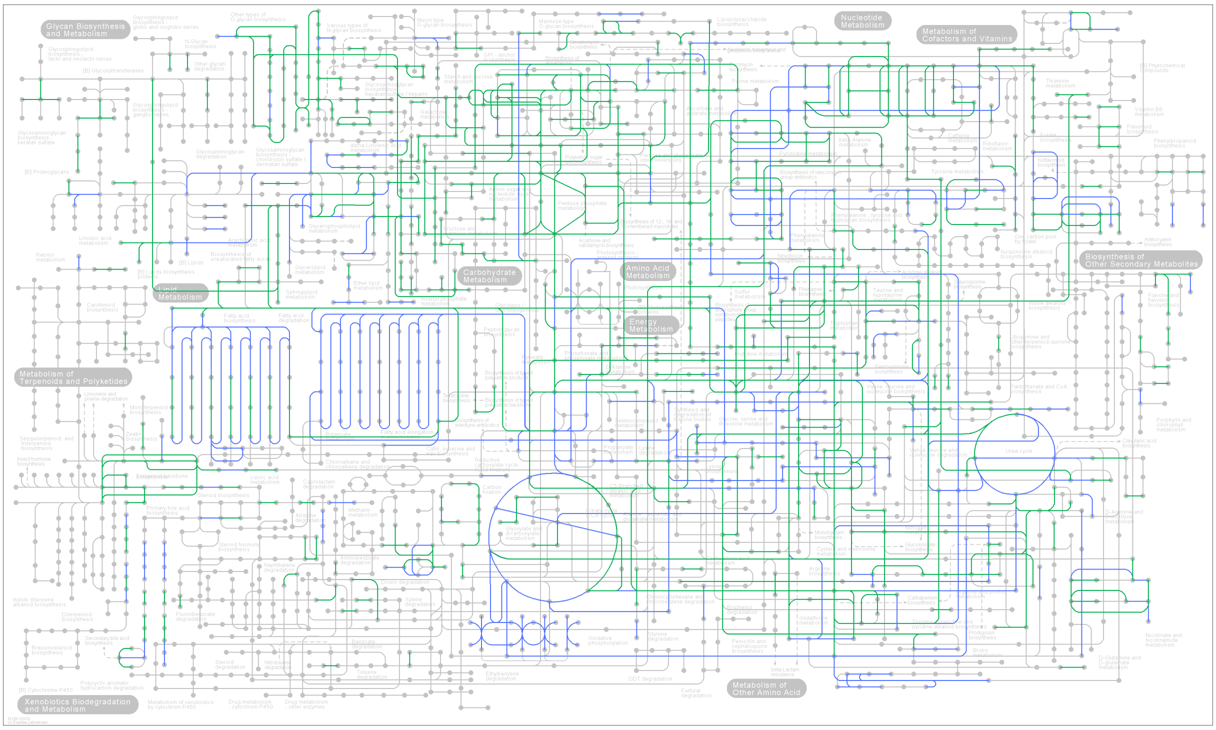

Supplementary data 2, Figure S12: KEGG map of cellular metabolism of *Go. avonlea* with the putative metabolic pathways in the mitochondrion highlighted in blue.

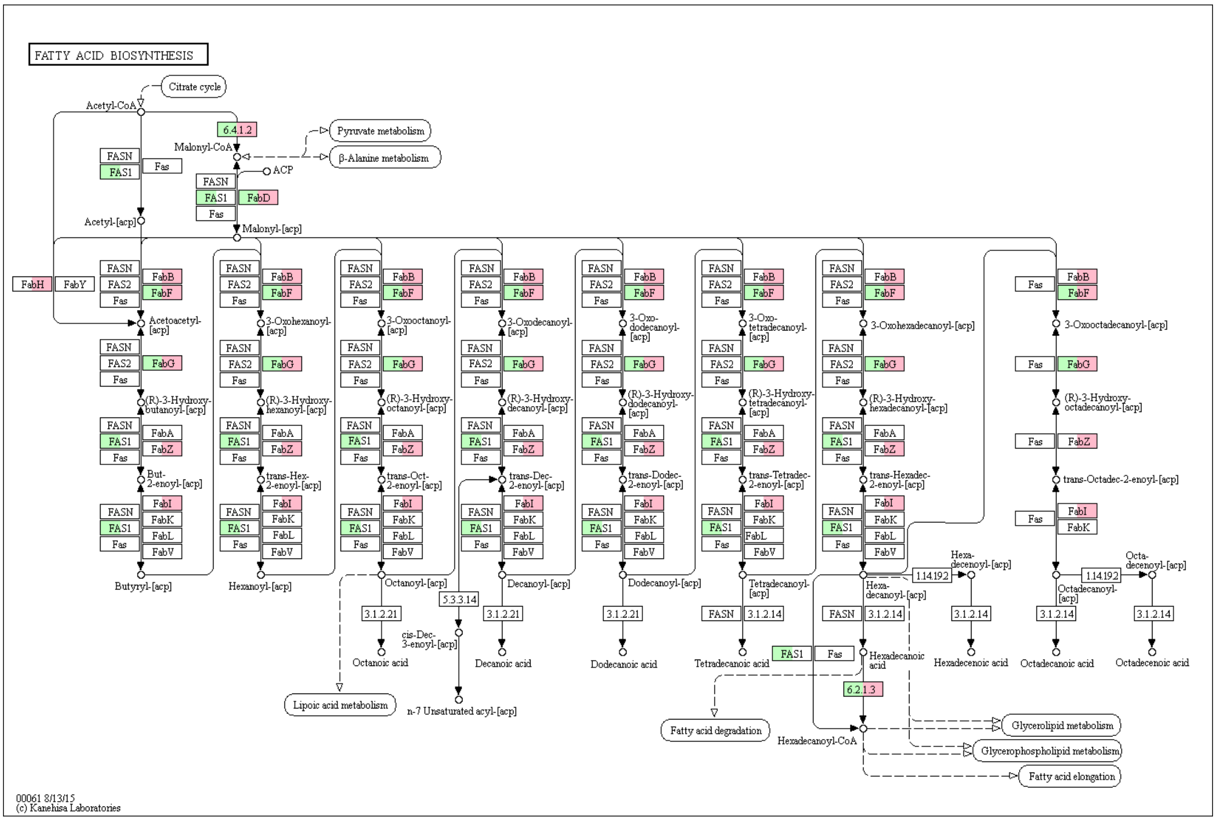

Supplementary data 2, Figure S13: KEGG representation of fatty acid biosynthesis. Green indicates the presence of a given protein in *Go*. *avonlea* while red indicates proteins found in *Gu*. *theta*. We observed that while both organisms possess a complete fatty acid biosynthesis pathway, it appears to be mitochondrial in *Go*. *avonlea* but plastid-localized in *Gu*. *theta*.

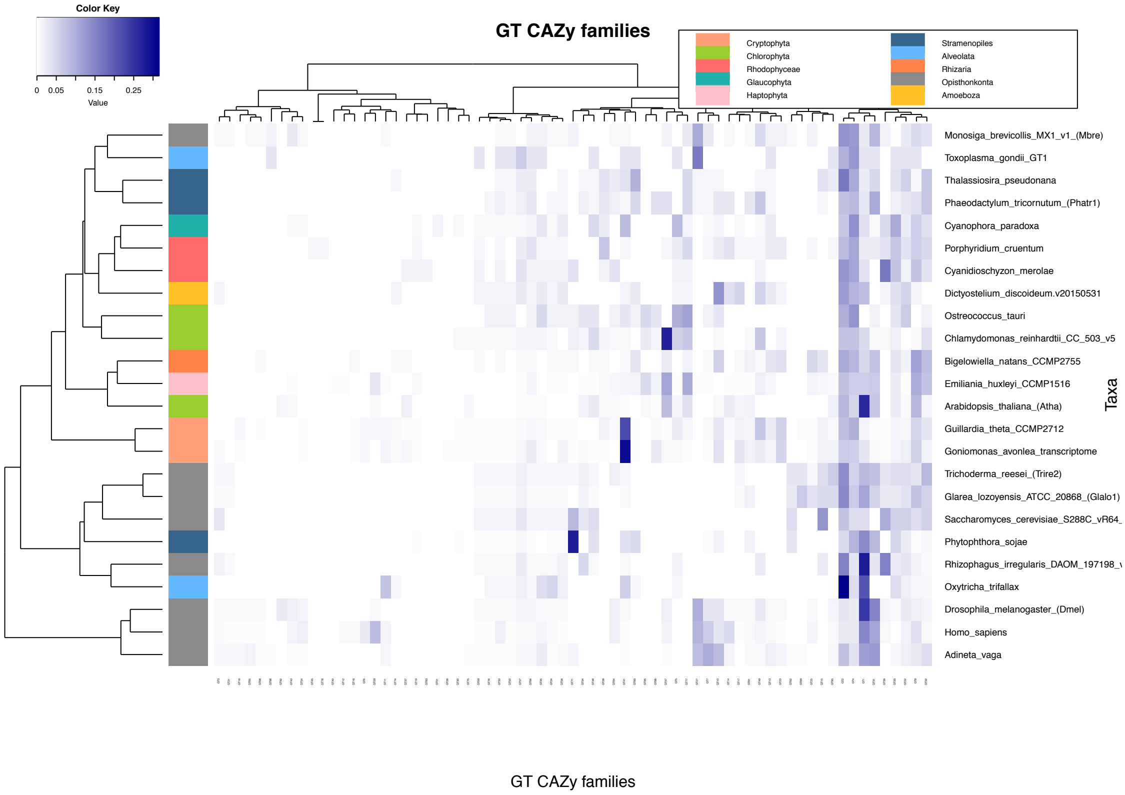

Supplementary data 2, Figure S14: Comparison of GlycosylTransferase (GT) CAZy families in *Go*. *avonlea* and other eukaryotes. The heatmap shows GT prevalence per eukaryote using a heat color scheme (white to blue), indicating low to high prevalence. Dendograms show the relative proximity of taxa on the left and the co-occurrence of CAZy families on the top. As expected, a close relationship between *Gu*. *theta* and *Go*. *avonlea* was observed. The analysis also shows that GT 41 is highly prevalent in these two organisms.

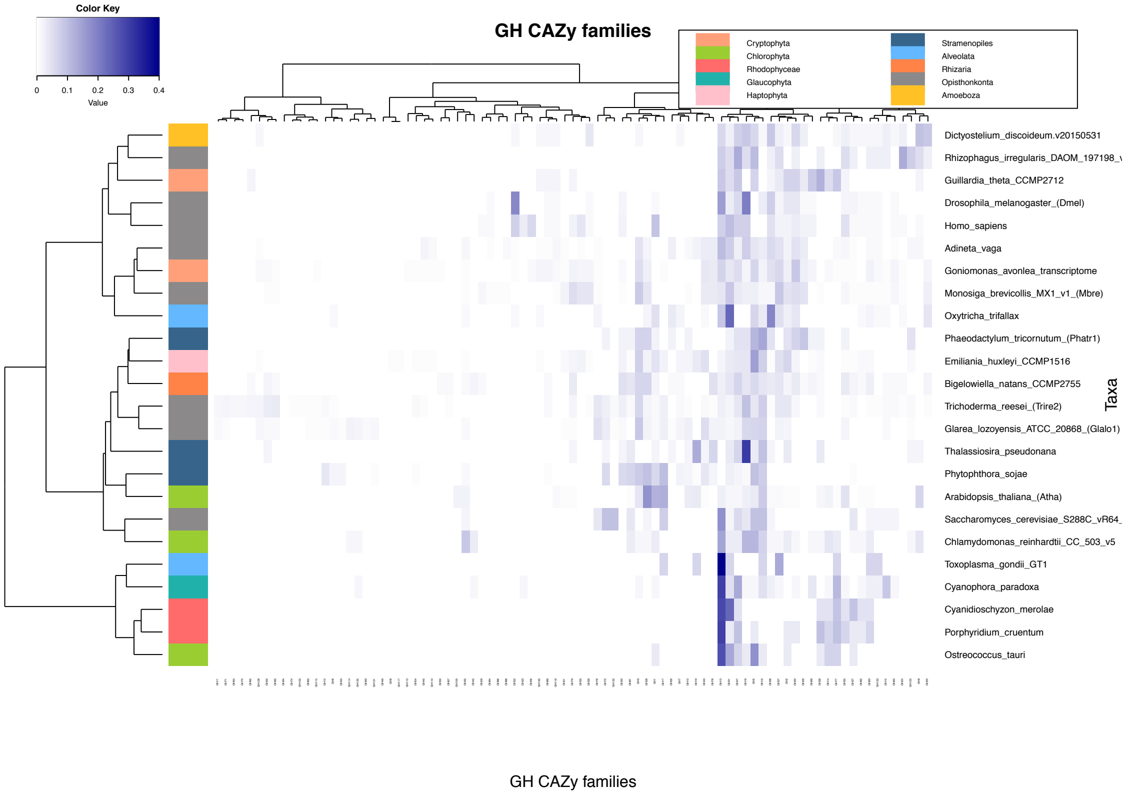

Supplementary data 2, Figure S15: Comparison of Glycoside Hydrolase (GH) CAZy families in *Go. avonlea* and other eukaryotes. The heatmap shows GH prevalence per eukaryote using a heat color scheme (white to blue), indicating low to high prevalence. Dendograms show the relative proximity of taxa on the left and the co-occurrence of CAZy families on the top. Surprisingly, we observed a close relationship between *Go. avonlea* and the rotifer *Adineta vaga*, and *Gu. theta* is also close to opisthokonts.

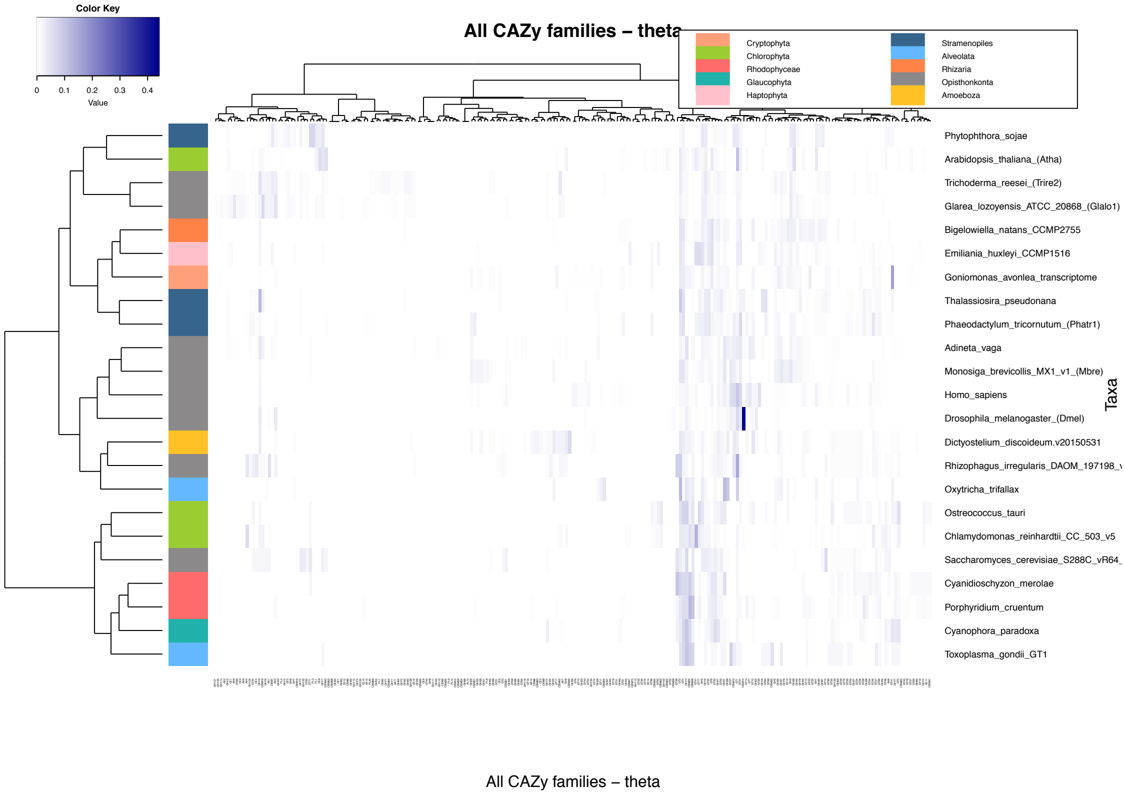

Supplementary data 2, Figure S16: Comparison of all CAZy families of *Go. avonlea* and other eukaryotes but with *Gu. theta* excluded from the analysis. The result suggests that the proximity between *Goniomonas avonlea* and algae derived from secondary plastid endosymbiosis is due to similar CAZy family profiles and perhaps similar physiologies.

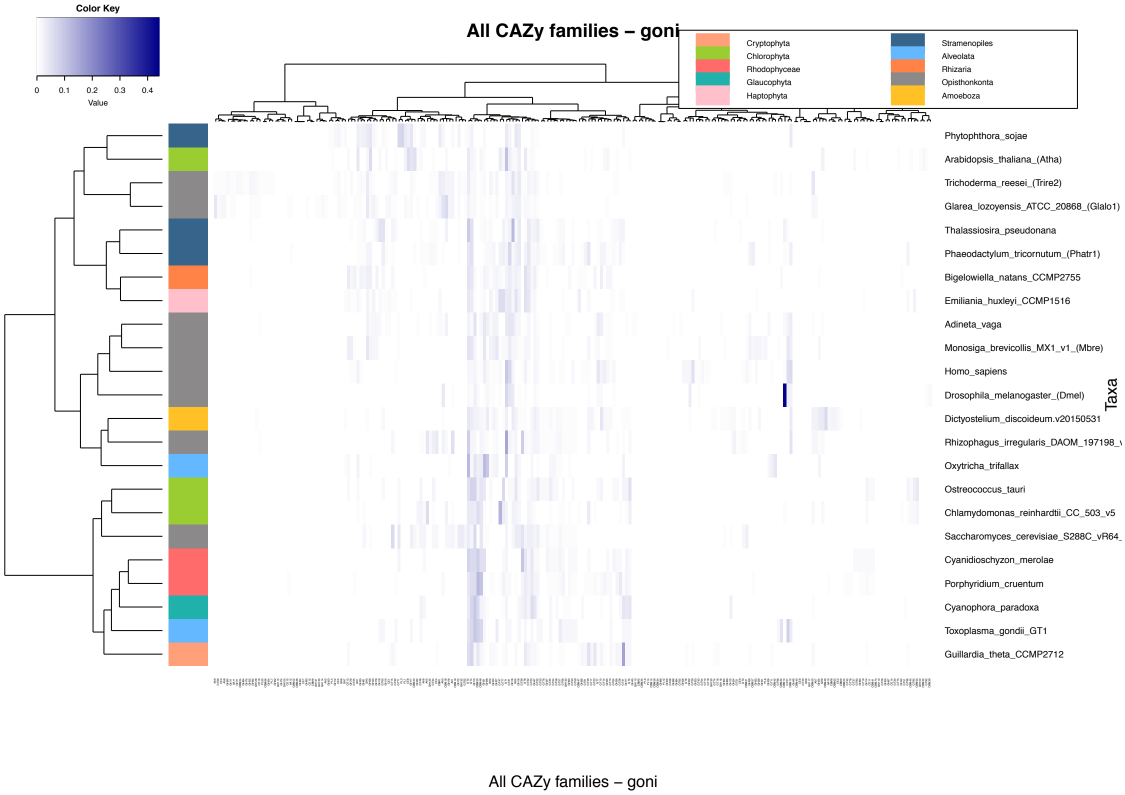

Supplementary data 2, Figure S17: Diagram showing a heatmap of CAZyme prevalence in each taxon (abundance within a particular CAZy family divided by the whole number of CAZy families predicted from the genome); the dataset is the same as that shown in Figure 5 but with *Go*. *avonlea* excluded. In this analysis *Guillardia theta* is closest to Rhodophyceae, Glaucophyta, and Chlorophyta, suggesting similar CAZy family profiles in each of these lineages.

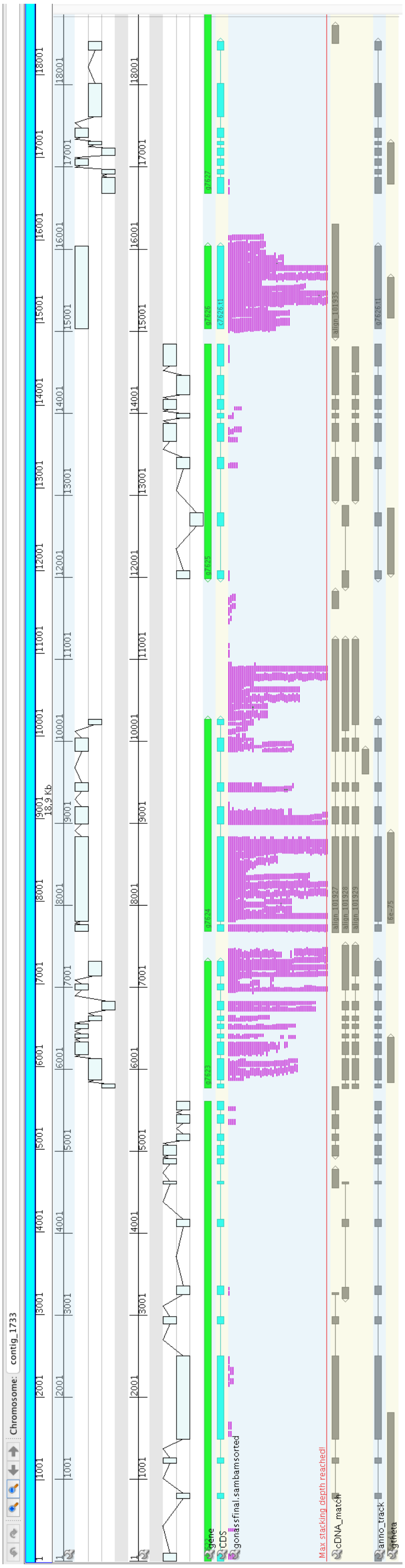

Supplementary data 2, Figure S18: Genome viewer showing gene models on contig 1733. We performed a top blast hit analysis for each of the predicted proteins, with the results as follows:
- g7622: Eukaryota, (id=48%, Evalue 2e-11), annotation SMC_N superfamily
- g7623: Bacteria (id=45%, Evalue 4e-65), annotation adh_short superfamily
- g7624: *Gu. theta* (id=34%, Evalue 5e-72), annotation PRK13609 (GT28)
- g7625: *Gu. theta* (id=39%, Evalue 7e-82), annotation Transpeptidase superfamily
- g7626: Eukaryota (id=40%, Evalue 7e-38), annotation SRPBCC superfamily
- g7627: Eukaryota (id=37%, Evalue 8e-37), annotation transient receptor potential cation channel subfamily

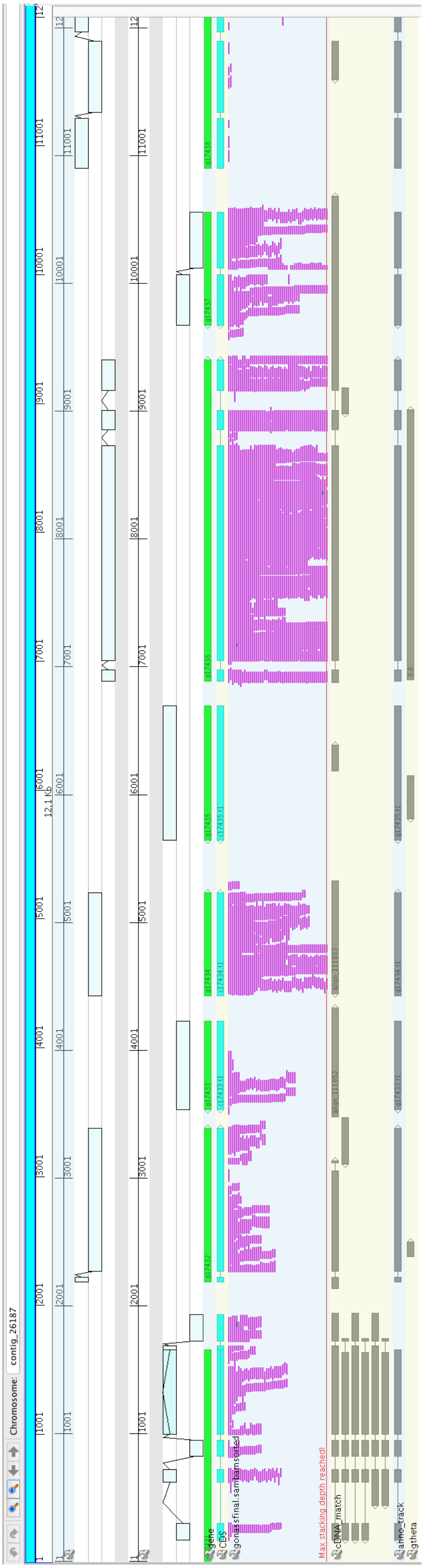

Supplementary data 2, Figure S19: Genome viewer from the Augustus website showing gene models from contig 26187. We performed a top blast hit analysis for each predicted protein. The results are as follows:

-g17431: Nitrospirales, CBM20 part of the GWD, g1 is a piece of the comp56169_c0, however Augustus did not predict the full GWD even with the cDNA sequence of the GWD

-g17432: *Drosophila yakuba* (id=37%, Evalue 0.006), annotation zf-RING_UBOX

- g17433: Eukaryota (id 25%, Evalue 6e-07), annotation AKAP7_NLS superfamily

- g17434: Eukaryota (id 27%, Evalue 4e-20), no annotation

- g17435: Eukaryota (id 32%, Evalue 1e-43), annotation Trm11 superfamily

-g17436: *Gu. theta* (id=83%, Evalue 0), annotation SunT (ABC transporter)

-g17437: Nothing

-g17438: Bacteria (id=43%, Evalue 4e-06), annotation LRR (leucine-rich repeat)
